# Dumbbell shaped structure loaded modified circular ring resonator based perfect metamaterial absorber for S, X and Ku band microwave sensing applications

**DOI:** 10.1038/s41598-024-56251-7

**Published:** 2024-03-07

**Authors:** Md. Golam Rabbani, Mohammad Tariqul Islam, Md. Moniruzzaman, Saeed Alamri, Abdullah Al Mahfazur Rahman, Asraf Mohamed Moubark, Md. Shabiul Islam, Mohamed S. Soliman

**Affiliations:** 1 Department of Electrical, Electronic and Systems Engineering, Faculty of Engineering and Built Environment, Universiti Kebangsaan Malaysia, 43600 Bangi, Selangor Malaysia; 2 Department of Electrical and Electronic Engineering, College of Engineering and Technology, International University of Business Agriculture and Technology, Uttara, Dhaka, 1230 Bangladesh; 3 Faculty of Engineering, Electrical Engineering Department, Al-Baha University, 65799 Alaqiq, Al-Baha Saudi Arabia; 4 Faculty Of Engineering (FOE), Multimedia University, Persiaran Multimedia, 63100 Cyberjaya, Selangor Malaysia; 5 Department of Electrical Engineering, College of Engineering, Taif University, P.O. Box 11099, Taif 21944, Saudi Arabia; 6 Department of Electrical Engineering, Faculty of Energy Engineering, Aswan University, Aswan, 81528 Egypt

**Keywords:** Metamaterial, Absorber, Transverse electric (TE), Transverse magnetic (TM), Microwave sensing, Materials science, Nanoscience and technology, Optics and photonics, Physics

## Abstract

In this paper, a new metamaterial absorber (MMA) is presented that exhibits peak absorptions at 3.26 GHz, 11.6 GHz, and 17.13 GHz within S, X, and Ku bands. The unit cell of the proposed MMA is constructed on an FR4 substrate having an electrical dimension of 0.144λ × 0.144λ, where wavelength, λ is calculated at the lowest absorption frequency. The unique structural design of the unit cell consists of two concentric copper rings with which dumbbell-shaped structures are attached. The rotating symmetrical structural design of this MMA provides around 93.8%, 96.47%, and 99.95% peak absorptance in the mentioned frequencies, which is invariable with the change of incident angle as well as polarization angle. The metamaterial properties of the proposed absorber are studied along with the surface current analysis. The MMA shows single negative behaviour and it also exhibits high-quality factors (Q factor) of 21.73, 41.42, and 51.90 at maximum absorptance frequencies. The MMA is analysed by it's equivalent circuit to understand the resonance phenomenon, which is verified through simulation in Advanced Design Systems (ADS) software. The testing is done on the developed prototype of the proposed MMA. Measurement results are in close proximity to the simulation results. Due to its high Q factor, high EMR, and insensitivity to polarization and angle of incidence, it can be utilized as a part of miniaturized microwave device. In addition, the proposed MMA can exhibit high sensing performance and flexibility to differentiate different oils in S, X, and Ku bands.

## Introduction

Metamaterials are artificially developed materials that have some extraordinary features like controlling and manipulating the behaviour of incoming electromagnetic signals^1^. Recently, these materials are getting more attention because of their rare characteristics like negative permittivity, permeability and refractive index, small size, and weight^2,3^. Due to these features, metamaterials are used in various applications like sensing^4^, detecting^5^, shielding^6^, absorbing^7,8^, and PV-based energy harvesting application^9–11^. Since the development of the first perfect metamaterial absorber in 2008^12^, the use of metamaterial in absorber design has gained significant interest, especially for applications in microwave and optical frequencies. The attraction towards metamaterial absorbers is increasing due to their low profile, perfect absorption capabilities and high performance^13–16^. Metamaterial absorbers can be used outside the microwave and optical spectrums. These absorbers can also be operated in infrared^17^ and terahertz applications^18^. Now, these days the use of single-band metamaterial absorbers is not frequent, whereas the growth of multiband absorbers is increasing rapidly. In this respect, the main concern is the performance of the absorber.

A multi-band metamaterial absorber is presented in^19^ for microwave uses. This modified split ring-based absorber exhibited a single negative characteristic at the resonance frequencies. Peak absorption of 99% at S and X bands can be achieved by the hexagonal-shaped modified split ring absorber^20^. Metamaterial absorbers in^21–23^ are deployed for energy harvesting purposes. The absorber described in^21^ is specifically engineered to span a broad frequency range of 1 GHz effectively. It achieves an exceptional absorption rate of 99.9% at a specific frequency of 5.5 GHz. The multilayer approach is adopted in^22^ to develop a pyramid structure absorber. The absorber of^23^ can provide wide-band operation for thermal-energy production purposes. The low-cost FR4 substrate-based metamaterial is used in^24,25^. A metamaterial absorber is presented in^26^, which is wide-band and insensitive to both polarization and broad angles. The structure is constructed based on a symmetrical design and uses surface mount resistors.The absorber has polarization-insensitive characteristics and achieves an absorptivity of over 80% over a wide variety of incident angles, up to 40°. Due to the asymmetric structure of the circular-shaped absorber^24^ it cannot provide a polarization-insensitive attribute. Near-perfect absorption of 99.7%, 99.9%, and 99.9% can be achieved by the triple band metamaterial structure of^6^. Harbinder et al., performed an evaluation of the sensing capabilities and absorbance of a wheel-shaped resonator featuring triangular spokes, with a particular emphasis on the metamaterial characteristics of the resonator. The absorber has absorption characteristics at a frequency of 10.42 GHz and demonstrates a high level of linearity in accurately detecting changes in moisture levels^27^. A very thin wide-band meta-surface polarization converter with two separate peaks at 11.66 and 18.13 GHz frequencies is presented by Roy et al. The structure is assessed using existing polarization converters and finds applications in the X, Ku and K bands^28^. However, due to the asymmetrical structure of the absorber, it cannot provide polarization-insensitive behaviour. Whereas the simple symmetrical structure of^25^ the absorber can provide polarization-insensitive characteristics covering the Wi-Fi frequencies with near-perfect absorption. Ranjan et al.^29^ is presented a new kind of Metamaterial Absorber (MA) that is insensitive to polarization and has a square form with four lumped resistors. It is designed for use in communications antennas, satellite technologies, and radar absorption materials. In^30^, a metamaterial absorber that is insensitive to polarisation is introduced. This absorber is designed to capture energy from Wi-Fi frequencies. The metamaterial absorber's structures for broadband applications are presented in^31–33^. A wide-band metamaterial cross-polarizer (MCP) configuration is designed for applications in the C and X bands^34^. The flexible sandwich-shaped metamaterial absorber of^31^ is capable of operating at sub 6G band and 5G applications whereas the flexible metamaterial absorber structure of^32^ can operate in Wi-Fi applications. In^35^, a triple-band metamaterial absorber is shown, which is not affected by polarisation and achieves absorption rates above 99% at frequencies of 8.11 GHz and 11.40 GHz, as well as over 96% absorption at a frequency of 15.12 GHz. This absorber has an ultra-thin structure and performs effectively at specific frequencies only. In^36^, a transparent and flexible metamaterial absorber is designed to cover 8–18 GHz frequencies for the optical application band. This absorber also has a low profile and is lightweight but can operate at a certain frequency. Thus, a significant scope is there to develop a metamaterial absorber to cover X and Ku bands for EM applications. The approach presented in^37^ for fabricating two wide-band metamaterial cross-polarizers is called advanced binary wind-driven optimisation (BWDO)^37^. Amir et al., present a Metamaterial ‘M.M.’ sensor that is specifically engineered to distinguish between pure and adulterated fuels and lubricants in X band region. Its objective is to enhance the detection of fuel adulteration through the utilisation of a compact sensor^4^. Ahasanul et al., present a metamaterial absorber for x and Ku band sensing applications in^38^. A sensor that senses pressure, temperature, density, and humidity utilises a metamaterial absorber and attains perfect absorption (PA) at frequencies of 6.46 GHz and 7.68 GHz^39^. It works only in the C band. In^40^, a flat metamaterial absorber based on the complementary Archimedes principle was designed and simulated for the purpose of refractive index sensing. The absorber exhibited significant absorption rates at different resonance frequencies. the model presented in^4,38–40^ have larger dimension which eventually execute low EMR value. Low EMR value can hamper the microwave application of the design. In addition, the absorption is also quite low at the resonance frequency. In many cases it is below 90% which could affect their absorption performances. The models of references^4,39,40^ offer sensing operation in single band whereas^38^ deals with double band sensing. So, it can be said that these designs have the lackings in sensing flexibility to operate in multiband.

The discussion mentioned earlier indicates that absorbers based on metamaterials have made their way into many microwave applications. Though there are many absorbers found in the literature, there is still room for further exploration in this area with unique structural design, compactness in dimensions, and application-oriented designs. The proposed MMA can provide the following distinctive characteristics: (1) a unique structural design that includes two circular rings in the resonating patch with which the dumbbell-shaped structures are attached, (2) the resonating patch is so designed that the structure is rotating symmetrically, which makes the absorber insensitive to polarization angle change as well as incident angle change, (3) the design provides three peaks of reflection coefficient at 3.26 GHz, 11.6 GHz, and 17.13 GHz frequencies that cover the S, X and Ku Bands with more than 90% absorbance, which makes it suitable for sensing and detecting applications, (4) sensing flexibility by operating in S, X and Ku bands, (5) high effective medium ratio (EMR). These novel features provide the advantages of implementation of the proposed MMA absorber in the miniaturization of microwave device for efficient absorption of unwanted microwave signals. In addition, the multiple absorption and sensing flexibility the absorber can be used in the liquid sensing application. The remaining sections of the paper are structured in the following manner: the description of the MMA unit cell design and simulation approach is provided in section “MMA design and simulation”. A rigorous parametric study based on various parameters is included in section “Parametric study”. However, the study of surface currents is conducted in section “Surface current analysis for the proposed MMA”, whereas the discussion of comparable circuit modelling is presented in section “Equivalent circuit modelling”. The sixth section examines at the results and compares the proposed MMA performance to other state-of-the-art methods. It focuses on the metamaterial characteristics of the proposed absorber, Section “Result and discussion” analyses the metamaterial characteristics of the suggested absorber, polarisation and incidence angle sensitivity of the structure, measured results, liquid sensing application and the proposed MMA’s performance compared to the state-of-the-art. The conclusion of section six presents key conclusions from many research and assessments of this MMA.

## MMA design and simulation

**Figure 1:**
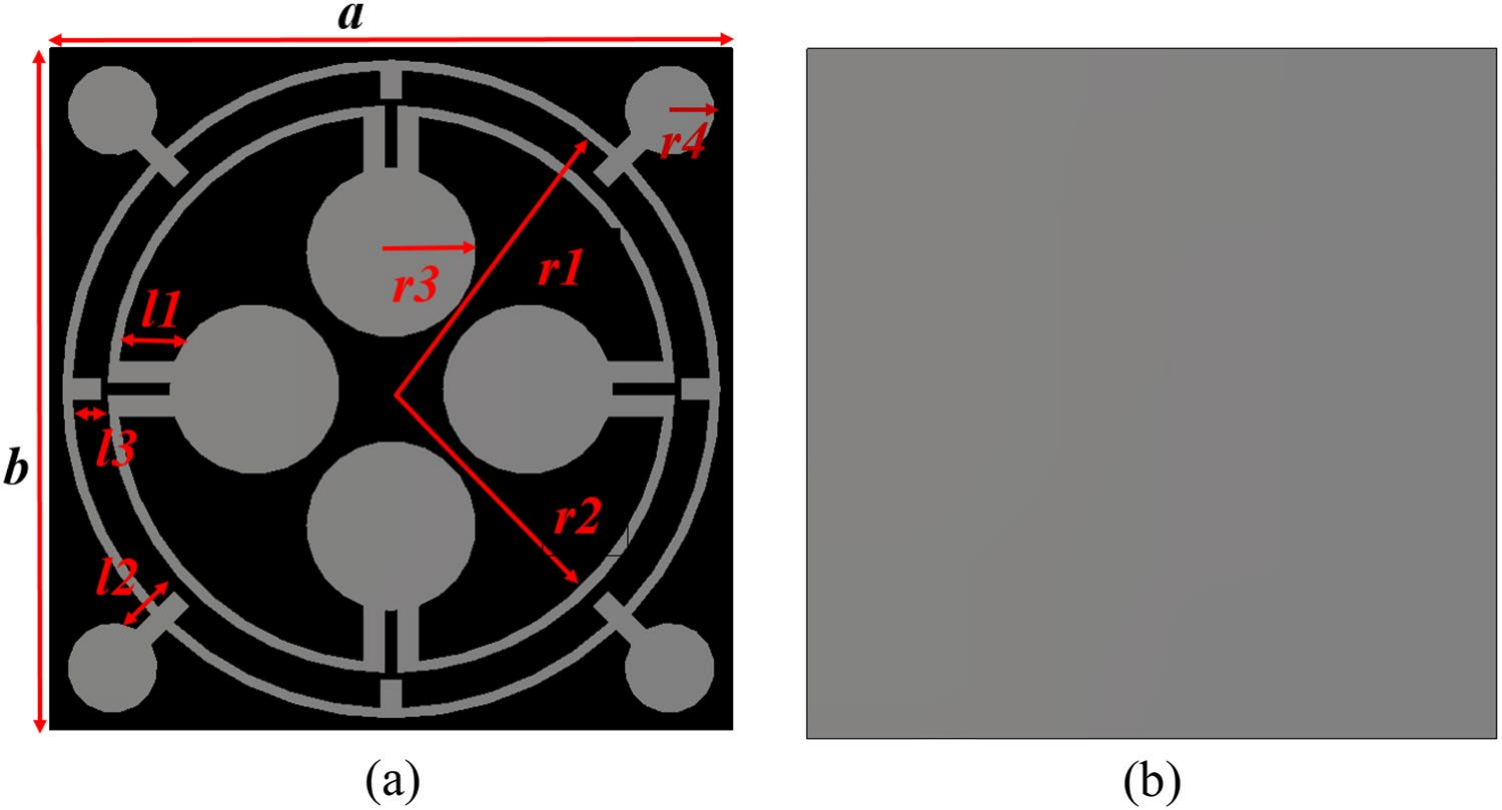
MMA unit cell: (**a**) front view, (**b**) back view.

The proposed MMA unit cell consists of a substrate, a resonator patch, and a metallic bottom, as shown in Fig. 1. The FR4 is a 1.6 mm thick substrate with a dielectric constant of 4.3 and a loss tangent of 0.02. On one side of this is a resonating patch, whereas on the opposite side, there is a full copper backplane. The copper backplane acts as a perfect reflector for the microwave; thus it hinders the signal to be transmitted from one side to the other side of the structure. The top resonating patch is so designed that it can create near-zero reflection at our targeted frequencies. Thus the incident wave on this MMA is trapped in between the top and bottom copper structures and eventually, absorbed by the substrate material. The overall dimension of the MMA unit cell is selected as 12 mm × 12 mm and the top resonating patch contains two circular rings each of them having 0.2 mm thickness. The distance of these rings from the centre is labelled as r1 and r2. Four dumbbell-shaped structures are attached to each ring as depicted in Fig. 1. The outer ring also contains four metallic stubs that are extended towards the centre. The proposed overall structure has rotational symmetry, allowing it to function as an ideal absorber. This is due to the presence of absorption processes in both transverse electric and transverse magnetic electromagnetic waves.

**Figure 2:**
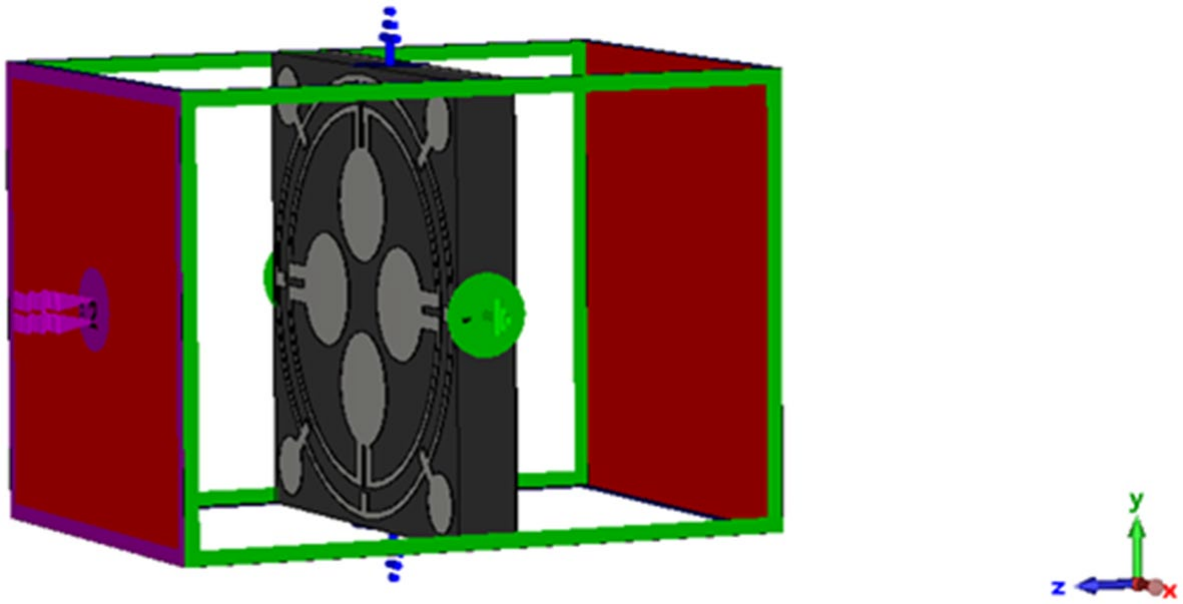
MMA cell simulation in CST microwave studio.

The dimensions of the various resonating patch sections are outlined in Table 1. The optimisation of these dimensions was accomplished by conducting multiple numerical simulations using the CST Microwave Studio Suite-2019. The simulation configuration is illustrated in Fig. 2. It consists of two waveguide terminals that are utilised to transmit and receive transverse electromagnetic waves over the frequency range of 2–18 GHz.

**Table 1:** List of the unit cell’s parameters.

Parameter	Dimension (mm)	Parameter	Dimension (mm)	Parameter	Dimension (mm)
*a*	12	*b*	12	*r1*	5.6
*r2*	4.8	*r3*	1.5	*r4*	0.8
*l1*	0.96	*l2*	0.96	*l3*	0.6

**Figure 3:**
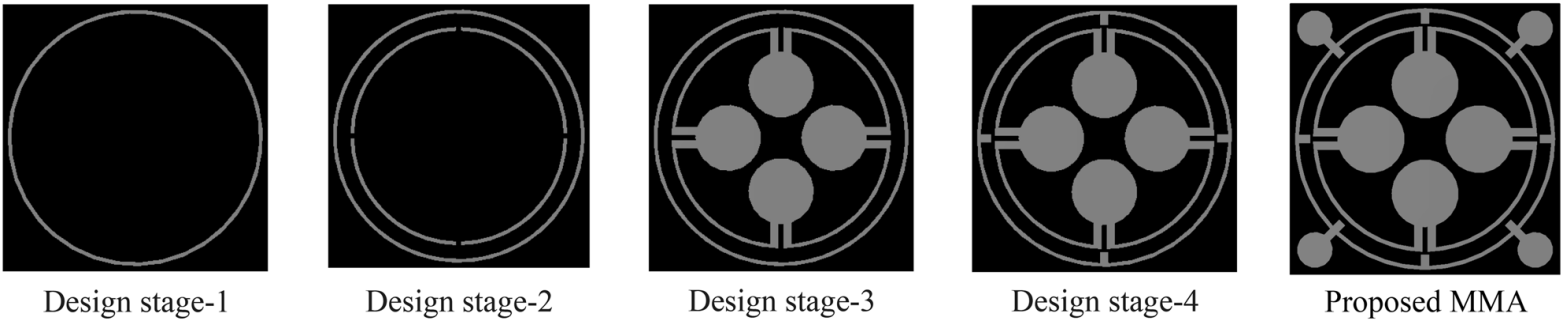
Design stage-1, Design stage-2, Design stage-3, Design stage-4 and the proposed MMA design of the proposed model.

The design is initiated with the inclusion of a copper ring in the resonating patch having a radius of 5.6 mm from the centre and a thickness of 0.2 mm as depicted in design 1 of Fig. 3. The absorption due to this ring is calculated by extracting reflection coefficient (S_11_) and transmission coefficient (S_21_) from simulations using Eq. (1)^41^.

1 $${\text{Absorption}},{\text{A }} = { 1} - {\text{S}}_{{{11}}}^{{2}} - {\text{S}}_{{{21}}}^{{2}}$$

The full copper backplane used with the design creates an obstacle for the transmission of the incident electromagnetic waves. The possible penetration of the electromagnetic wave through a conducting surface can be determined using the term skin depth which can be calculated using the Eq. (2)^42^.

2 $${\text{Skin depth}}, \, \delta \, = \sqrt {\frac{\rho }{\pi \mu f}}$$

**Figure 4:**
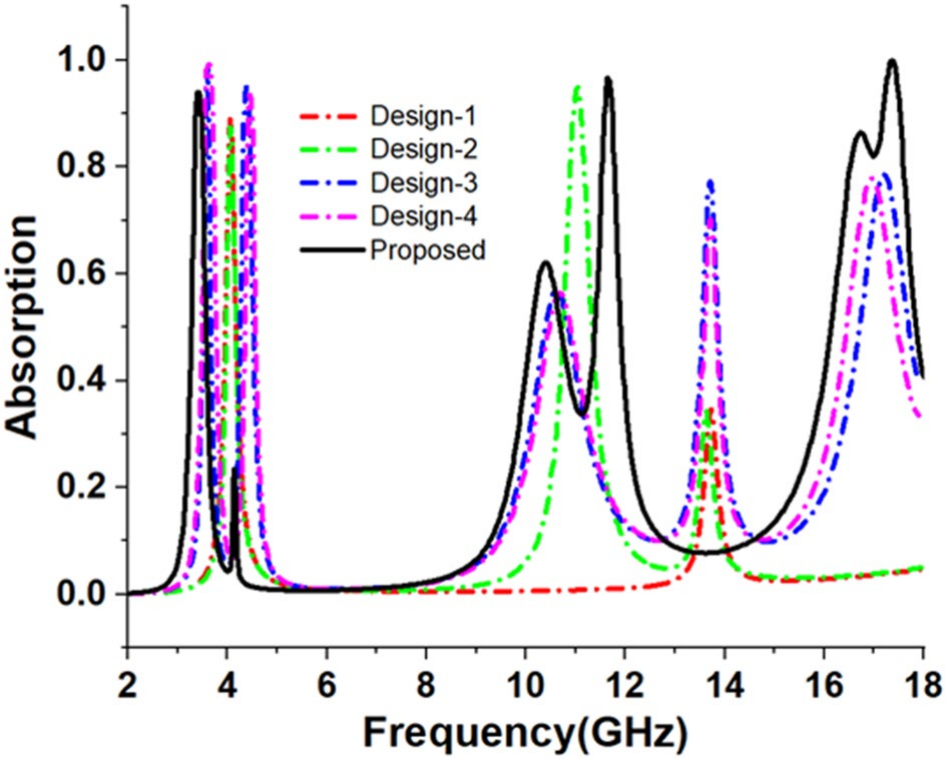
Absorptivity for several design processes leading to the suggested unit cell.

A copper backplane with a thickness of 0.035 mm is adequate to block the transmission of electromagnetic waves. Since transmission becomes zero, Eq. (1) can be written as:$A = 1 - S_{11}^{2}$. The obtained absorption spectrum for design 1 of Fig. 3 is presented in Fig. 4. From Fig. 4, it is noticed that the outer circular ring provides a peak absorption at 4.06 GHz having 89% absorption Subsequently, an additional circular split ring is incorporated within the initial ring illustrated in design 2 of Fig. 3. The incorporation of this ring induces an additional resonance of S11 with a magnitude of 0.23 at 10.99 GHz, thereby aiding in the achievement of 95% absorption at this level of frequency. Due to the nearly unchanged absorption peak at 4.05 GHz, two bands are responsible for two absorption peaks. As depicted in design 3 of Fig. 3, the design is further modified by the addition of four circular metallic stubs near the split gap of the second ring. The alteration is evident in the displacement of the peak absorption frequencies that were present in the preceding stage. In this design step, two major absorption peaks are obtained at 3.54 GHz and 4.45 GHz with a magnitude of 98.8% and 95.6% respectively. Three other minor absorption peaks are noticed around 10.56 GHz, 13.8 GHz, and 17.23 GHz. Design 4 incorporates four metallic stubs attached to the outer ring. Peak absorption frequencies are reduced by the coplanar capacitances that form between these extended metallic stubs and the inner ring. A drastic shift in resonances was noticed when the dumbbell-shaped structures are attached at four corners of the outer ring (as shown in the proposed MMA of Fig. 3). This inclusion causes a drastic change in electromagnetic interaction and near-perfect impedance matching between the MMA structure and free space occurs around 3.26 GHz, 11.6 GHz, and 17.13 GHz. As a result, peak absorption of about 93.8%, 96.47%, and 99.95% is attained in these frequencies that are classified as belonging to the S, X, and Ku bands, respectively. Table 2 provides a summary of the data obtained for various design processes.

**Table 2:** Comparison of design stages with respect to absorptance peaks and application frequency bands.

Different design steps	Maximum absorbance frequency (GHz)	Highest absorption (%)	Bands covered
Design-1	4.077, 13.787	89.03%, 34%	C, Ku-band
Design-2	4.077, 11.02, 13.61	87.21%, 94.75%, 33.73%	C, Ku-band
Design-3	3.58, 4.38, 10.92, 16.928	97.8%, 94.5%, 99.7%	S, X, Ku-band
Design-4	10.384, 16.72	48.7%, 74.94%	X, Ku-band
Proposed design	3.26, 11.6, 17.13	93.8%, 96.47%, 99.95%	S, X, Ku-band

The narrowband and significant absorption levels at three frequencies 3.26, 11.6, and 17.13 GHz show the frequency selectivity of the absorber. Half-power maximum bandwidths (HMBW) of the proposed MMA are150 MHz, 280 MHz, and 330 MHz for the above-mentioned peak absorptions respectively. The sensitivity is calculated using the formula in Eq. (3), which yields the Q factor,

3 $${\text{Q }} = f_{0} /{\text{HMBW}}$$

where *f*_0_ is indeed the frequency where the maximum absorption in a particular band is achieved. The Q factors for such frequencies in the proposed MMA are found to be 21.73, 41.42, and 51.90, respectively. Furthermore, the MMA cell has an off-resonance absorption compared with fewer than 20%. The suggested MMA’s great selectivity so demonstrates its potential to be used in perceiving and detecting operations.

## Parametric study

This section evaluates the absorption qualities of the proposed MMA by varying the size of various parts of the resonating patch, the height of the copper backplane, and the thickness of the substrate material. For the variation in parameter, MMA impedance changes significantly; thus, the matching of this impedance with the free space impedance is hampered, which affects peak absorption as well as peak absorption frequencies.

### Effects of change of radius of four outer dumbbell-shaped resonators

**Figure 5:**
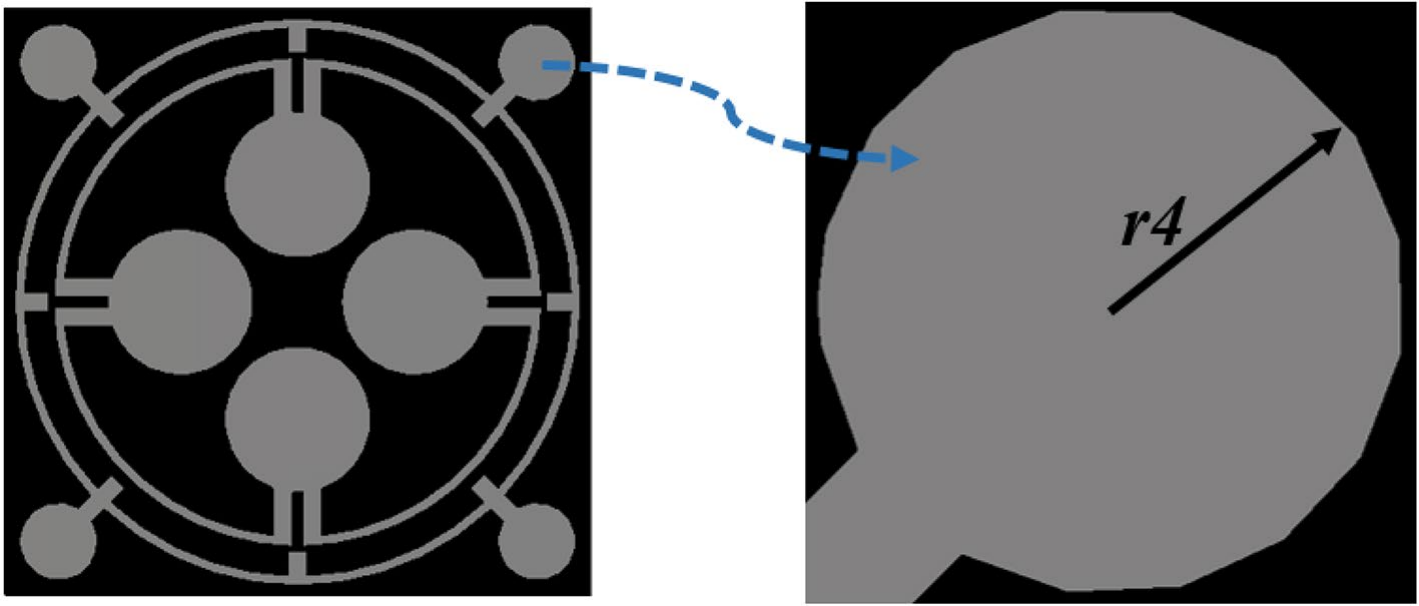
Enlarged view of dumbell shaped structure attached with the outer ring.

**Figure 6:**
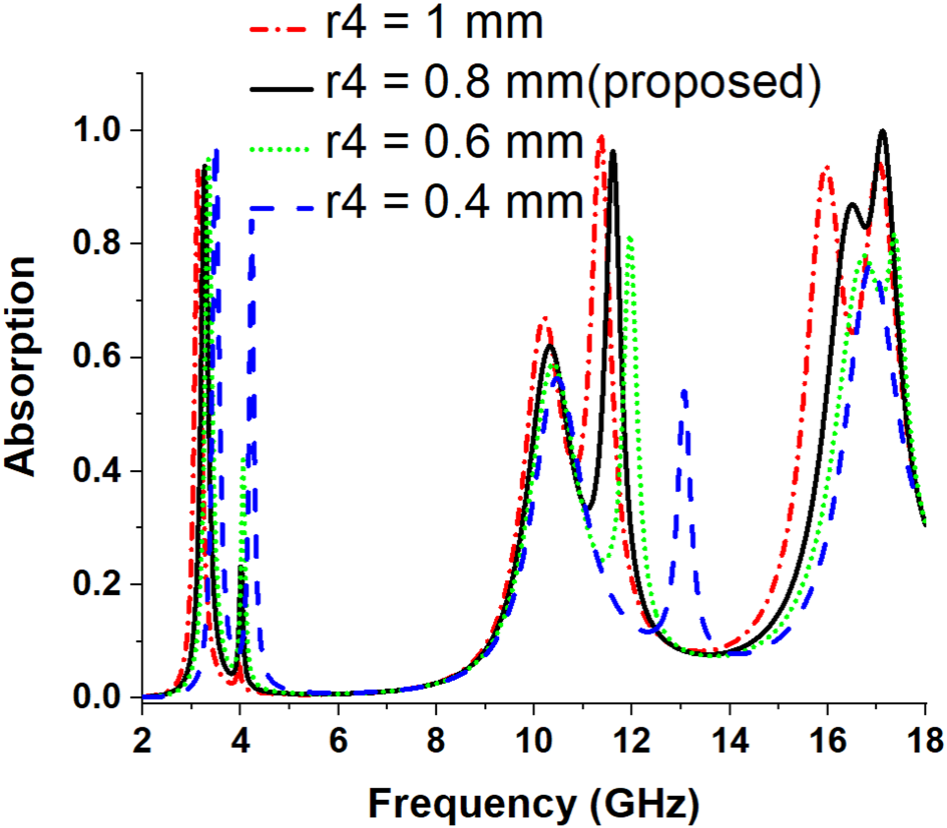
Absorption spectrums for variation in radius of outer dumbbell-shaped resonators.

In this study, the radius of the dumbbell-shaped structures attached to the outer ring is modified, and its effect on the absorption phenomena is studied. Figure 5 illustrates the enlarged view of this structure, and the radius (r4) of each dumbbell attached to the outer ring is changed simultaneously at an equal step of 0.2 mm. The variation in the radius of the outer dumbbell-shaped resonating part of the absorber has an impact on the absorption level and the corresponding resonance frequencies. The data of peak absorptions and the corresponding resonance frequencies are given in Table 3, and the absorption spectrums are plotted in Fig. 6. From Fig. 6, it is noticed that with the decrease in radius, the peak absorptions and their corresponding resonance frequencies are shifted to the right side.

**Table 3:** Absorption for variation in radius of outer dumbbell-shaped resonators radius.

Radius (mm)	Peak absorption frequency (GHz)	Peak absorption (%)
***r4*** = 0.4	3.48, 4.22, 10.48, 13.05, 16.91	98%, 84%, 56%, 54%, 76%
***r4*** = 0.6	3.33, 4.05, 10.38, 11.95, 16.77, 17.38	94%, 41%, 59%, 81%, 77%, 81%
***r4*** = 0.8	3.26, 11.6, 17.13	93.8%, 96.47%, 99.95%
***r4*** = 1	3.12, 10.19, 11.34, 15.96, 17.05	92%, 67%, 99%, 93%, 94%

### Effect of variation in radius of inner dumbbell-shaped resonators

**Figure 7:**
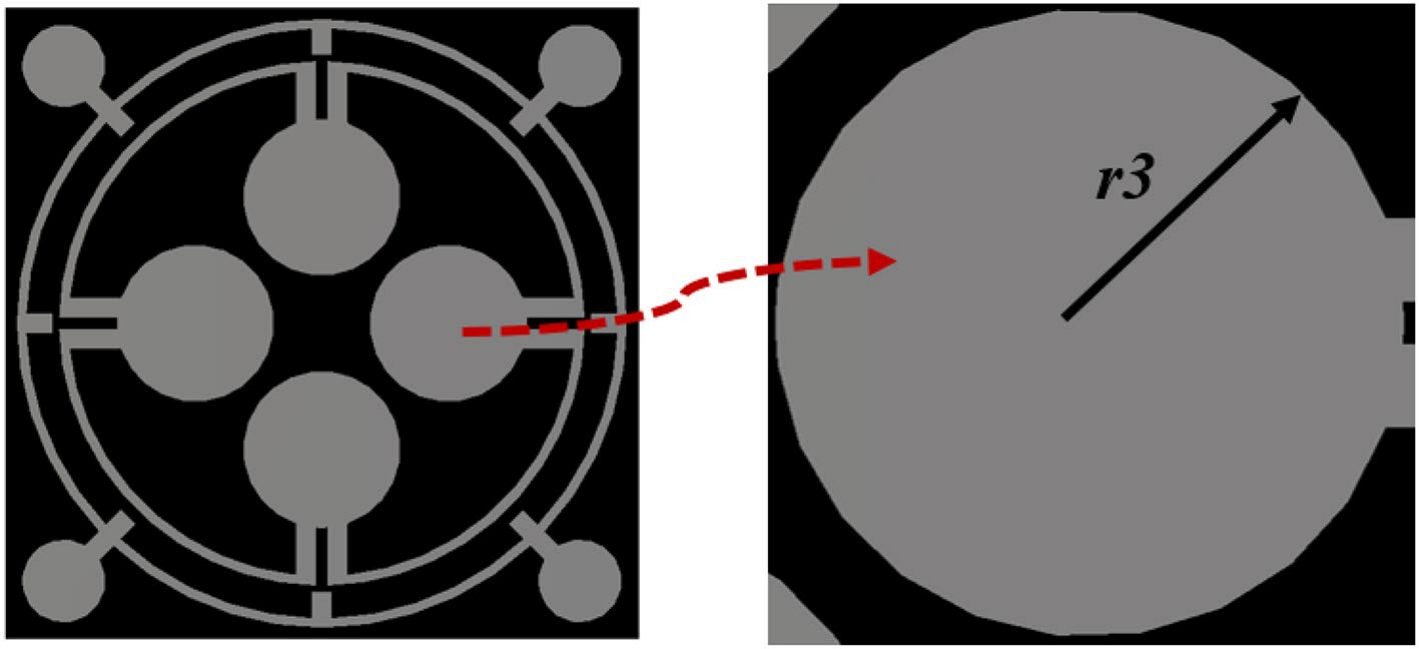
Enlarged view of dumbbell shaped structure attached with the inner ring.

**Figure 8:**
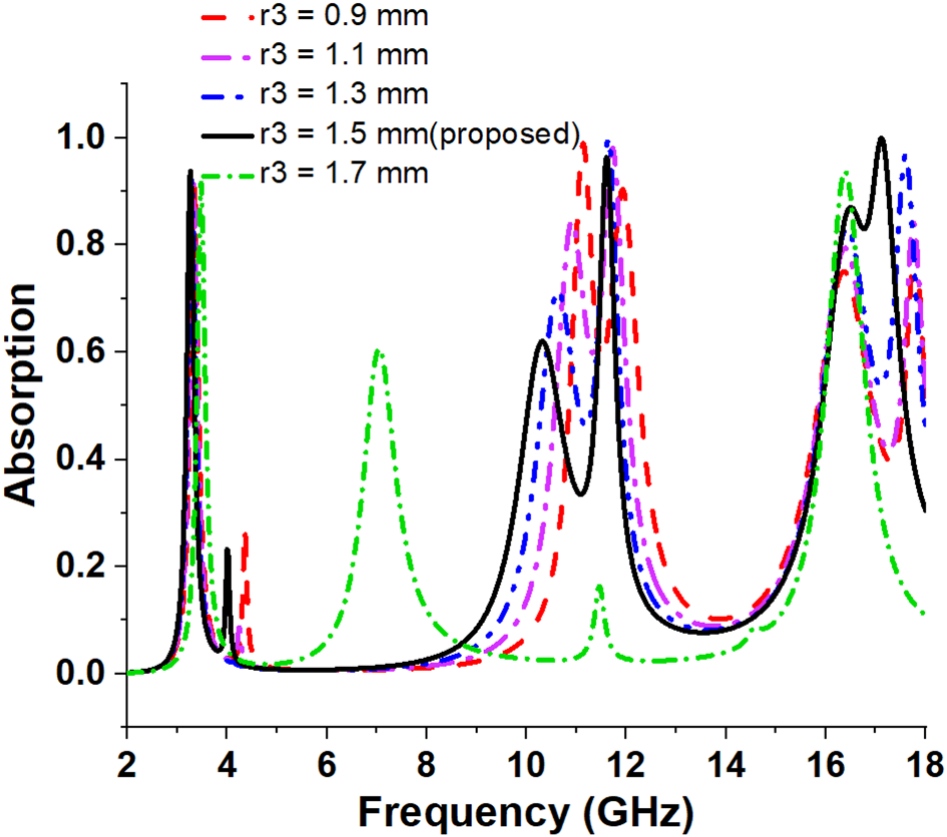
Absorption spectrums for variation in radius of inner dumbbell-shaped resonators.

An enlarged view of an inner dumbbell-shaped structure is depicted in Fig. 7, and an investigation is made by changing the radius *r3* of four such structures simultaneously. The values of r3 are modified from 1.1 to 1.7 mm, with a uniform increment of 0.2 mm. The resulting peak absorptions and their related resonance frequencies are shown in Table 4. Additionally, the absorption spectrums are graphically represented in Fig. 8. An analysis of the data presented in Table 4 expresses that the change of r3 has minimal impact on the first resonance (around 3.26 GHz) and the corresponding absorption level of the absorber. However, the change of *r3* exhibits a significant impact on the second and third resonances and their corresponding absorption levels, which is obvious from the data presented in Table 4 as well as the plots depicted in Fig. 8, indicating that these dumbbell-shaped structures’ dimensions have played a vital role for peak absorption and corresponding frequency modulations at high and medium frequencies. As the dimension increases, the mutual coupling effect between these structures increases, which shows an impact on peak absorption frequencies as well as on the peak absorptions.

**Table 4:** Absorption for variation in radius of inner dumbbell-shaped resonators radius.

Radius (mm)	Maximum absorbance frequency (GHz)	Highest absorption (%)
***r3*** = 0.9	3.33, 11.13, 11.93, 16.38, 17.78	90%, 98%, 90%, 75%, 79%
***r3*** = 1.1	3.31, 10.91, 11.72, 16.38, 17.76	91%, 84%, 98%, 79%, 84%
***r3*** = 1.3	3.28, 10.63, 11.62, 16.42, 17.6	92%, 71%, 99%, 83%, 96%
***r3*** = 1.5 (proposed)	3.26, 11.6, 17.13	93.8%, 96.47%, 99.95%
***r3*** = 1.7	3.47, 7.05, 16.38	91%, 60%, 93%

### Effects of change of length of four inner extended rectangular metallic stubs

**Figure 9:**
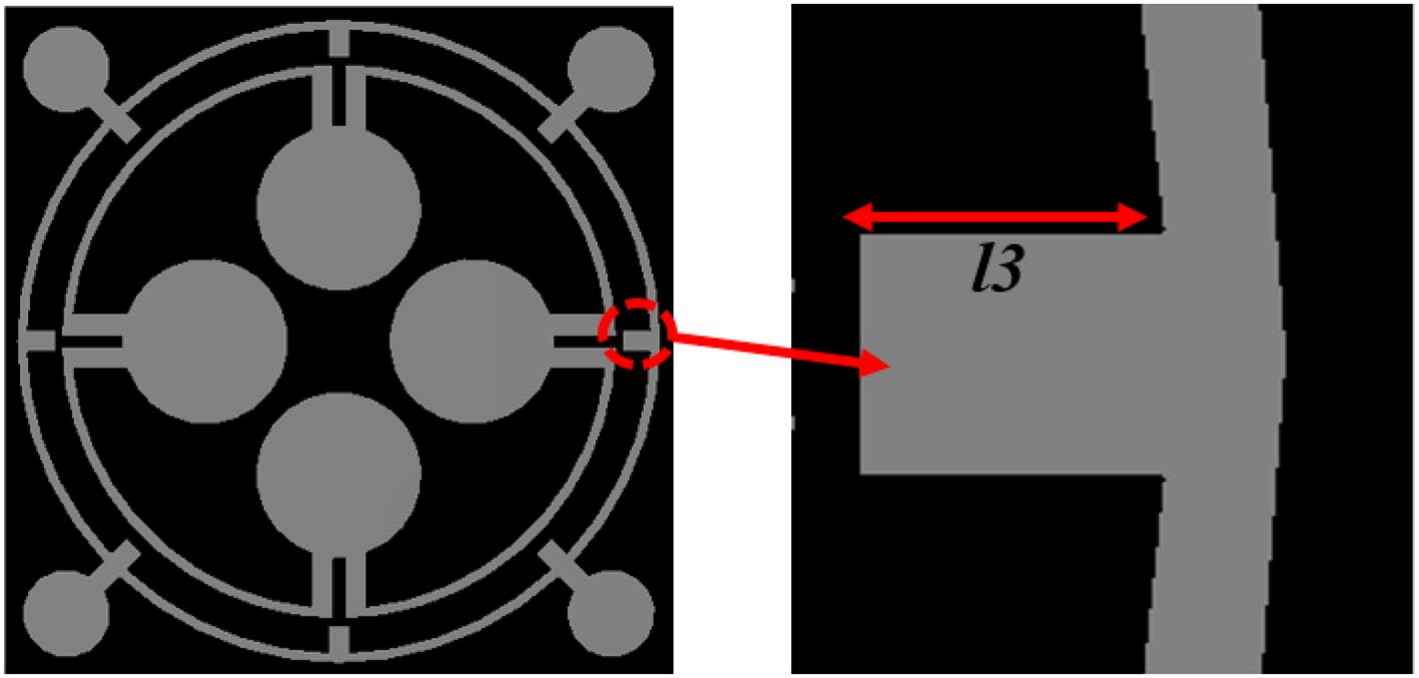
An enlarged view of the extended metallic stubs of the outer ring.

**Figure 10:**
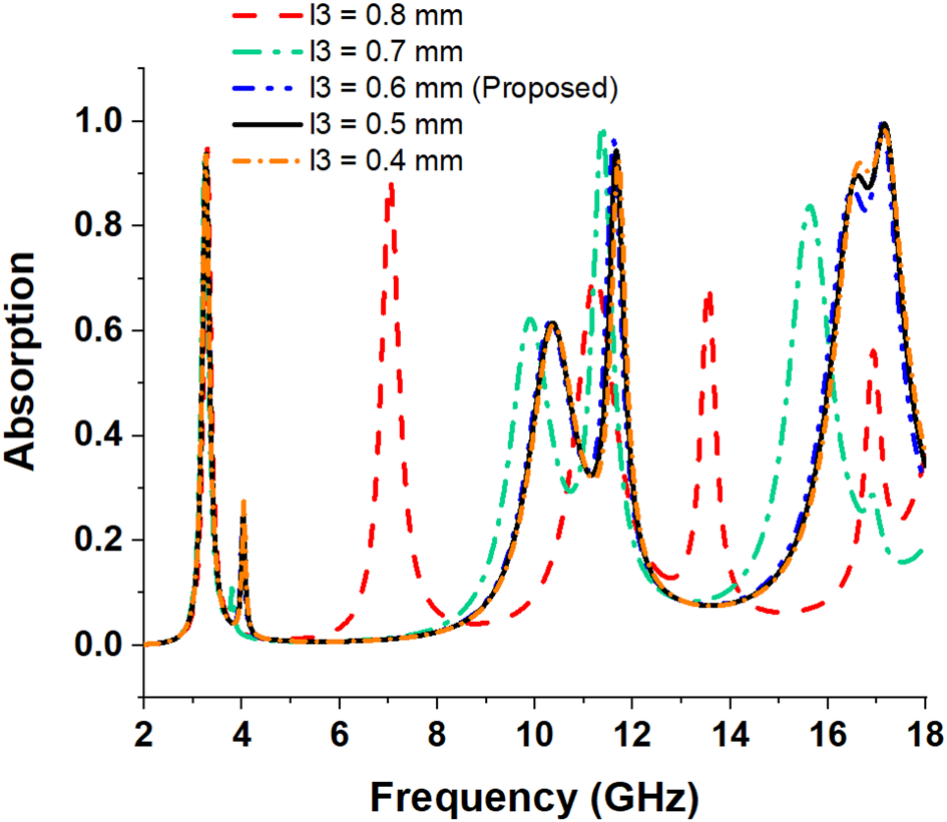
Absorption spectrums for various length of inner extended rectangular metallic stubs.

An enlarged view of the extended metallic stub attached to the outer ring is shown in Fig. 9. This section shows the effects of altering the length of four inner extended rectangular metallic stubs. The peak absorption levels and the corresponding resonance frequencies are given in Table 5, and the absorption spectrums are drawn in Fig. 10. For *l3* = 0.4 mm, a comparatively wider bandwidth of 0.78 GHz from 16.54 to 17.32 GHz is achieved with more than 90% of the absorption level. When *l3* is changed to 0.7 mm, the second resonance of the MMA shifts to 11.37 GHz to increase the absorption by 99%. A drastic shift of the third absorption frequency to 15.61 GHz with a reduced absorption having a magnitude of 83% is also noticed at this length. A close investigation revealed that when *l3* is 0.7 mm, the extended metallic stubs become very close in proximity to the inner ring. Consequently, a small distance separating the two rings results in an amplified capacitance, which modifies the upper resonance frequency in a downward direction. It is observed that a maximal absorption of 89% is attained at a frequency of resonance of 7.02 GHz when l3 = 0.8 mm. Moreover, two other resonances are obtained at 13.55 GHz and 16.92 GHz with low absorption peaks of 68% and 56%, respectively. Noteworthy to mention that when *l3* = 0.8 mm, peak absorption decreases gradually, which is obvious from the data presented in Table 5. The resonance around 3.26 GHz is nearly unaffected at this length. The reason for this abrupt change in absorption phenomena is that when *l3* is 0.8 mm, two rings become interconnected with each other. Thus, the total resonating patch acts as a single LC circuit with a dominant absorption at 3.27 GHz, having a peak absorption of 94%. From this study, it can be concluded that with the help of these length changes, the frequencies near the targeted resonance frequencies can be tuned to provide a high absorption level.

**Table 5:** Absorption for various lengths of inner extended rectangular metallic stubs.

Length (mm)	Peak absorption frequency (GHz)	Peak absorption (%)
*l3* = 0.4	3.25, 11.71, 17.17	93%, 92%, 98%
*l3* = 0.5	3.26, 11.65, 17.16	93%, 93%, 99%
*l3* = 0.6 (Proposed)	3.26, 11.6, 17.13	93.8%, 96.47%, 99.95%
*l3* = 0.7 mm	3.24, 11.37, 15.61	94%, 99%, 83%
*l3* = 0.8 mm	3.27, 7.02, 11.25, 13.55, 16.92	94%, 89%,70%, 68%, 56%

### Effects of the thickness change of substrate material

**Figure 11:**
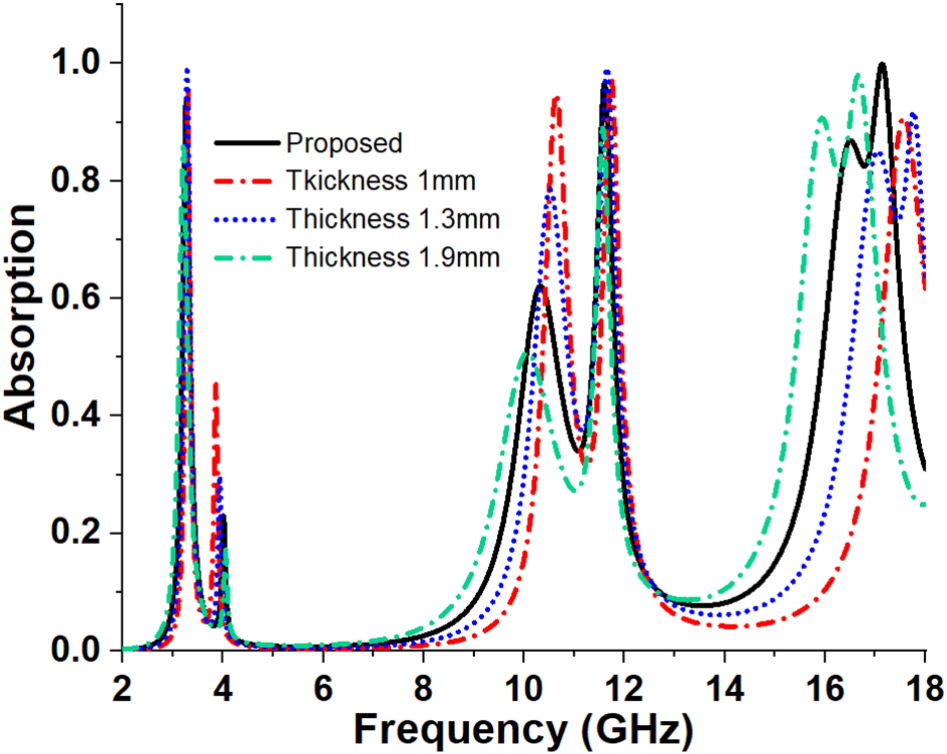
Absorption for varied substrate thickness variations.

In this section, the effect of FR4 substrate thickness change is analysed. The data of peak absorption at corresponding resonance frequencies for different thicknesses of substrate is presented in Table 6, and the absorption spectrum is plotted in Fig. 11. A comparison is made between the frequency deviation and peak absorption of the proposed MMA model and the variation of substrate thickness observed for four distinct values of substrate thickness, t. As the thickness, t increases from 1 to 1.9 mm, the resonance frequencies are observed to shift from the right to the left, as shown in Fig. 9.

**Table 6:** Absorption for various thicknesses of substrate material.

Thickness,* t* (mm)	Peak absorption frequency (GHz)	Maximum absorption (%)
*t* = 1.0	3.29, 10.65, 11.72, 17.56	95.72%, 94.6%, 97.8%, 90.8%
*t* = 1.3	3.28, 10.56, 11.65, 17.02, 17.76	99.53%,77.93%, 99.28%, 85.1%, 91.8%
*t* = 1.6 mm (Proposed)	3.26, 10.35, 11.6, 16.51, 17.13	93.8%, 62%, 96.47%, 86.89%, 99.95%
*t* = 1.9	3.2, 10.06, 11.56,15.92, 16.65	85.77%, 50%, 88.99%, 92.06%, 98%

### Effects of the copper backplane height change

**Figure 12:**
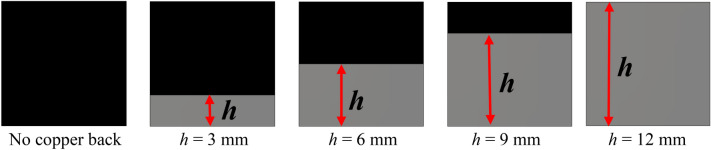
Back view of the propsed MMA with various hieght of the copper backplane.

**Figure 13:**
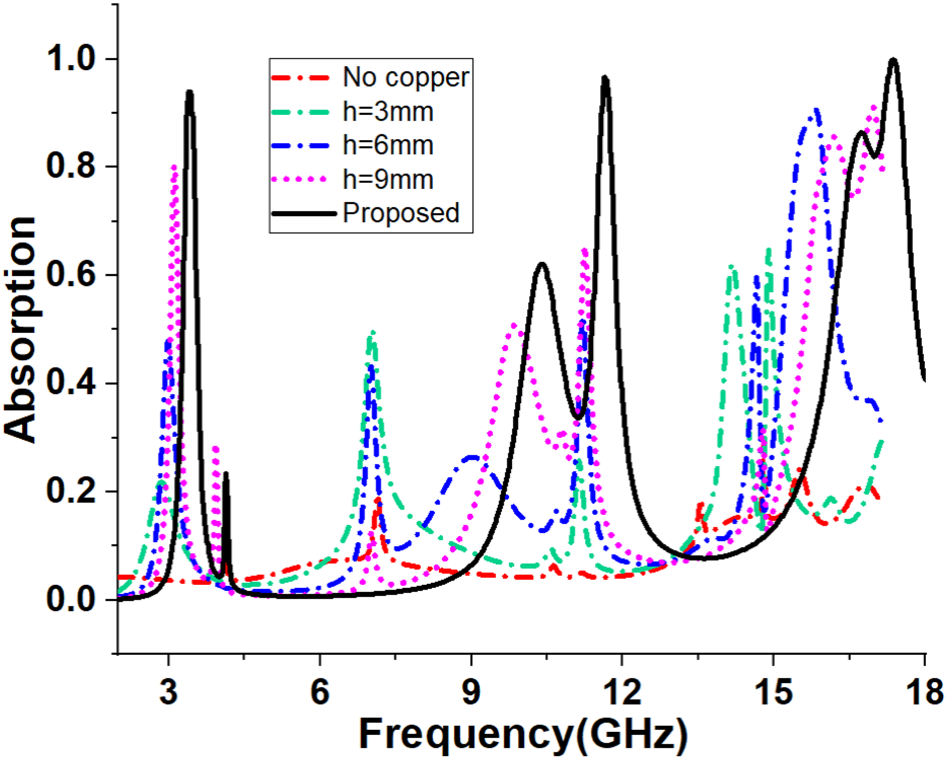
Spectrums of absorption for varied backplane.

An investigation is made to understand the effect of the copper backplane on the absorption of the proposed MMA. Figure 12 presents the copper backplane from no copper to full copper back with an increasing height (*h* = 3 mm, 6 mm, 9 mm) of the copper. The absorption spectra for different heights of the copper back are depicted in Fig. 13 which indicates that the level of absorption is increased for the continuous enhancement of backplane height. From the graph of Fig. 13, it is noticed that when no copper backplane is used, the absorption is almost zero in most of the frequencies in the investigation range. The highest absorption in this condition is nearly 20%. Absent of copper back causes the transmission of the signal as there is no metallic barrier in the backside of the MMA to hinder the flow of wave energy. Absorption increases steadily with the height of copper within the backplane. This increasing absorption is obtained due to the trapping of the incident wave between the copper back and top resonator. Transmission progressively decreases as the height of the copper back increases, as the copper’s thickness is greater in comparison to the the skin depth of the transmitted wave. Thus, the wave is trapped inside the substrate layer and eventually absorbed. The transmission of an electromagnetic wave becomes null when a full copper backplane is utilised, resulting in the complete reflection of the wave. Absorption in this circumstance is determined entirely by the reflection coefficient. The resonating patch is designed in such a way that the reflection coefficient becomes near zero at 3.26 GHz, 11.6 GHz, and 17.13 GHz, and electromagnetic wave within the substrate material is absorbed. Thus peak absorption of 93.9%, 96.65%, and 99.99% at 3.26 GHz, 11.6 GHz, and 17.13 GHz respectively are noticed in Fig. 13 for the full copper backplane.

## Surface current analysis for the proposed MMA

**Figure 14:**
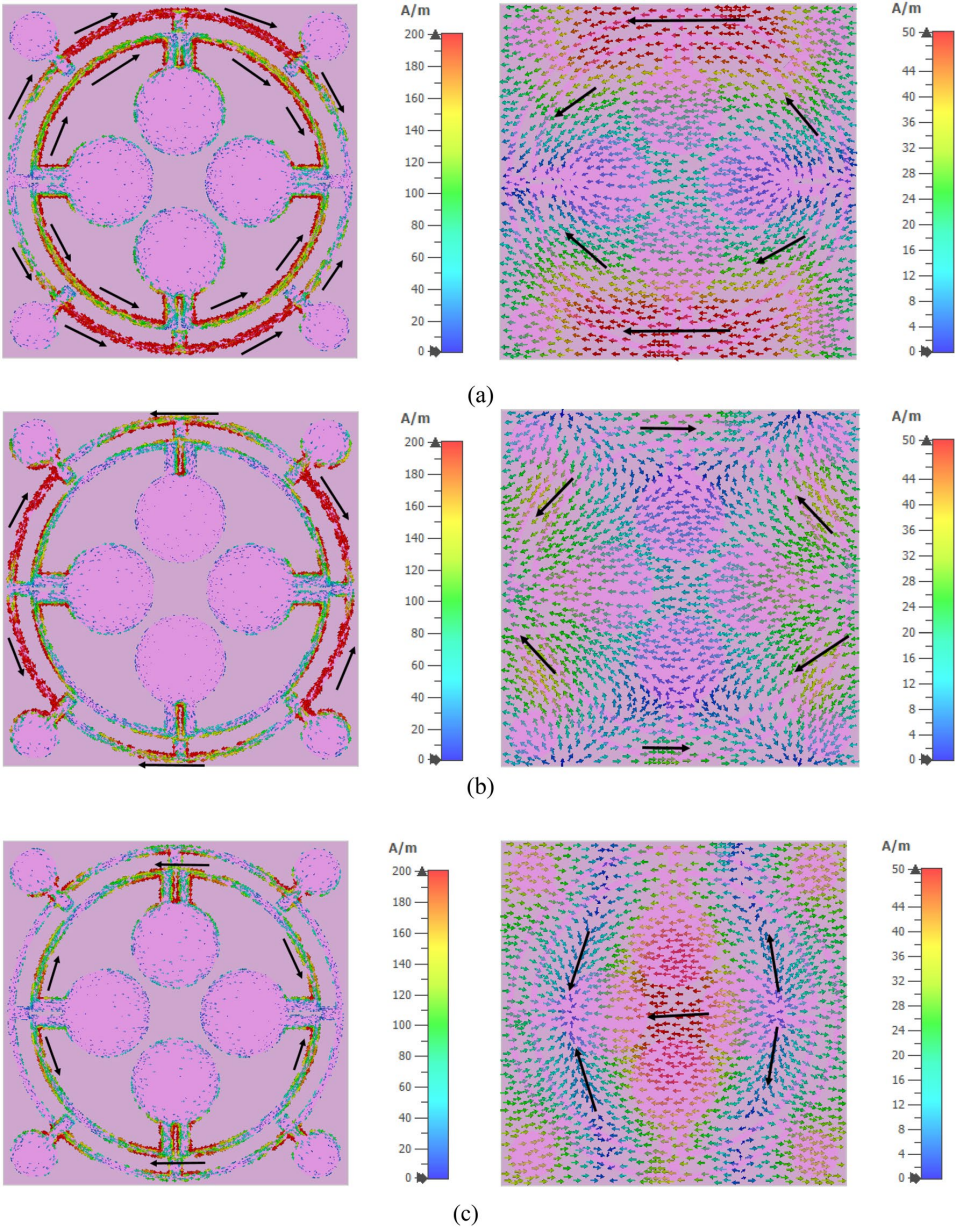
Surface current distribution of the proposed absorber at: (**a**) 3.26 GHz, (**b**) 11.6 GHz and (**c**) 17.13 GHz.

The surface current of the proposed MMA is investigated in this section for three resonance frequencies of 3.26 GHz, 11.6 GHz, and 17.13 GHz. In Fig. 14a the front and back surface current distributions at 3.26 GHz are presented. A strong current distribution is observed at the upper and lower parts of the most outer ring resonator of the absorber on this frequency. The direction of the current is clockwise for the upper part and anticlockwise for the lower part of the outer circle resonator. It is also noticed that a significant amount of the clockwise current is also flowing through the upper inner part of the inner circular ring resonator and a similar amount of current is flowing in the opposite direction through the lower inner part of the inner circular ring resonator. Although the density of the current is low on the innermost surface, it is considered in the linked point which is shown in Fig. 14a. The direction of the current on those portions of the back surface is opposite to the front surface current. As a result, magnetic dipole resonance arises from a current loop caused by the currents that are flowing antiparallel in the front and rear layers^43^. For this reason the resonance peak occurs at 3.26 GHz. At 11.6 GHz, the direction of the current reversed for the upper and lower parts of the outer ring resonator with a significantly reduced magnitude which is shown in Fig. 14 (b). Meanwhile, the existence of a significant amount of surface current is observed at the left and right half of the outer ring resonator clockwise and antilock wise as shown in Fig. 14b whereas the current distributions on those sections of the back surface become reversed. At this frequency, due to these antiparallel currents, a magnetic dipole is created which eventually formed the resonance for peak absorption. At 17.13 GHz, the concentration of current becomes high at the joining segments of an upper and lower inner circle of the resonator here the direction is anticlockwise for the upper segment and clockwise for the lower segment as shown in Fig. 14c. In addition, a significant amount of current is observed at the joining segments of the left and right inner circles of the resonator. From both left and right joining sections two different directional currents are produced one is moving in clockwise and another is moving in the anticlockwise direction. The backside currents of these sections are opposite to the currents of the front sides. As a result, the resonance at 17.13 GHz is produced because of the antiparallel current in the front and back planes.

## Equivalent circuit modelling

**Figure 15:**
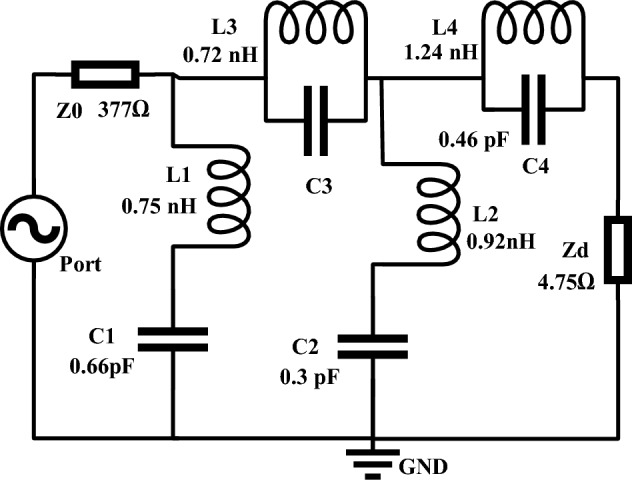
Equivalent circuit of the MMA unit cell.

The proposed MMA is analysed in terms of its equivalent circuit, as depicted in Fig. 15, considering the resonance effect of the various segments of the MMA cell as exhibited in CST 3D simulation.

Metallic strips and split gaps can generate a metamaterial inductor and capacitor, making a resonant LC tank circuit at a frequency computed using Eq. (7)^44^:

4 $${\text{f }} = { }\frac{1}{{2{\uppi }\sqrt {{\text{LC}}} }}$$

L and C represent, respectively, the inductance and capacitance of the structure. By employing Eq. (8), the capacitance between the splits can be computed.

5 $${\text{C }} = {{ \varepsilon }}_{0} {\upvarepsilon }_{{\text{r}}} { }\frac{{\text{A }}}{{\text{d }}}\left( {\text{F}} \right)$$

The strip area is represented by A, the inter-strip distance is denoted by d, and ε0 and εr indicate the absolute and relative permittivities of the medium, respectively. Within the framework of transmission line theory, the equivalent inductance can be determined by using Eq. (9)^45^.

6 $${\text{L}}\left( {{\text{nH}}} \right) = { }1.257{ } \times { }10^{ - 3} {\text{ a }}\left[ {{\text{ln}}\left( {{ }\frac{{\text{a}}}{w + t}{ }} \right){ } + { }0.078} \right]{\text{Kg}}$$

Kg = 0.57 0.145ln The correction factor is denoted as w/h in an equation where t represents the mean radius of a circle, w signifies the width of a microstrip line, and w′ and h′ signifies the depth and breadth of the substrate, respectively.

**Figure 16:**
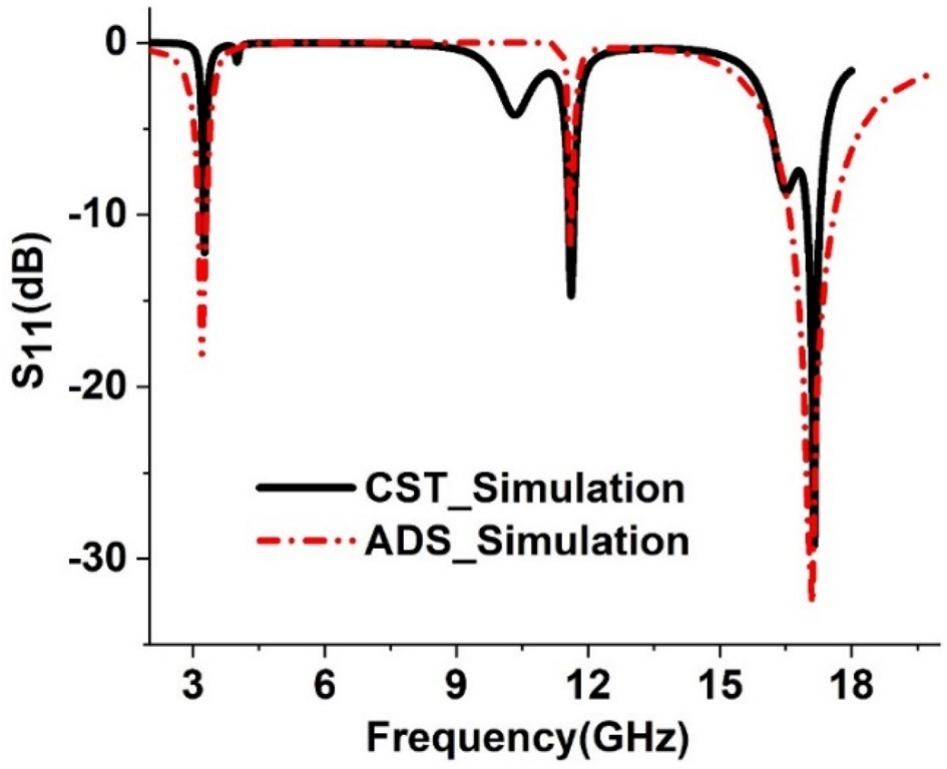
Performance comparison between S_11_ obtained in ADS and CST.

In this case, the inductor represents two circular rings, capacitor pairs L1, C1, and L2, C2. L3 and C3 are considered the mutual inductance and capacitances between the two rings. Whereas mutual effects among the innermost dumbbell-shaped structure are represented by the inductor-capacitor pair L4, C4. Since the substrate layer absorbs the energy, its effect is represented by impedance Zd. On the other hand, Z0 represents free space impedance. The circuit is designed in an Advanced Design System (ADS), and component values are finalized by tuning the component values. Initial values for the capacitors are considered 1 pF and each inductor is 1 nH. Then, the inductance and capacitance values are tuned and S_11_ values are compared with the same obtained in CST, and component values are finalized when the resonances have occurred in the vicinity of the peak absorption frequencies. S_11_ graphs obtained in ADS and CST are presented in Fig. 16. From this Figure, it is found that both plots show the close similarity for resonances of S_11,_ indicating that the proposed RLC resonance circuit represents the same resonance phenomena of the MMA unit cell. Thus, the circuit components' values are determined, and the circuit is validated. Through the tuning process is noticed that the dip of S_11_ resonances can be controlled by the substrate impedance Zd. As the impedance increases from a lower value, it is seen that resonance dip at frequencies 3.2 GHz and 17.12 GHz increases with the increasing values of Zd, and maximum dip is obtained when the impedance is 4.75Ω. Since a strong mutual coupling effect has existed among the different segments, for this reason, it is noticed from the circuit that every RC branch shows its impact on all three resonances.

## Result and discussion

In this section, simulated and measured results are compared and analysed. Firstly, the metamaterial behavior is studied by extracting the effective values of permittivity and permeability. From the permittivity and permeability data, impedance is calculated, and the dependence of absorption is studied. The effects of the TE and TM modes’ signal on the MMA performance are also studied in this section, along with changing the incident and polarization angle variation. The measured result is also analysed in this section in comparison to the simulated result. A comparison is also performed with some recent works on metamaterial-based absorbers.

### Metamaterial behaviour of the proposed design

**Figure 17:**
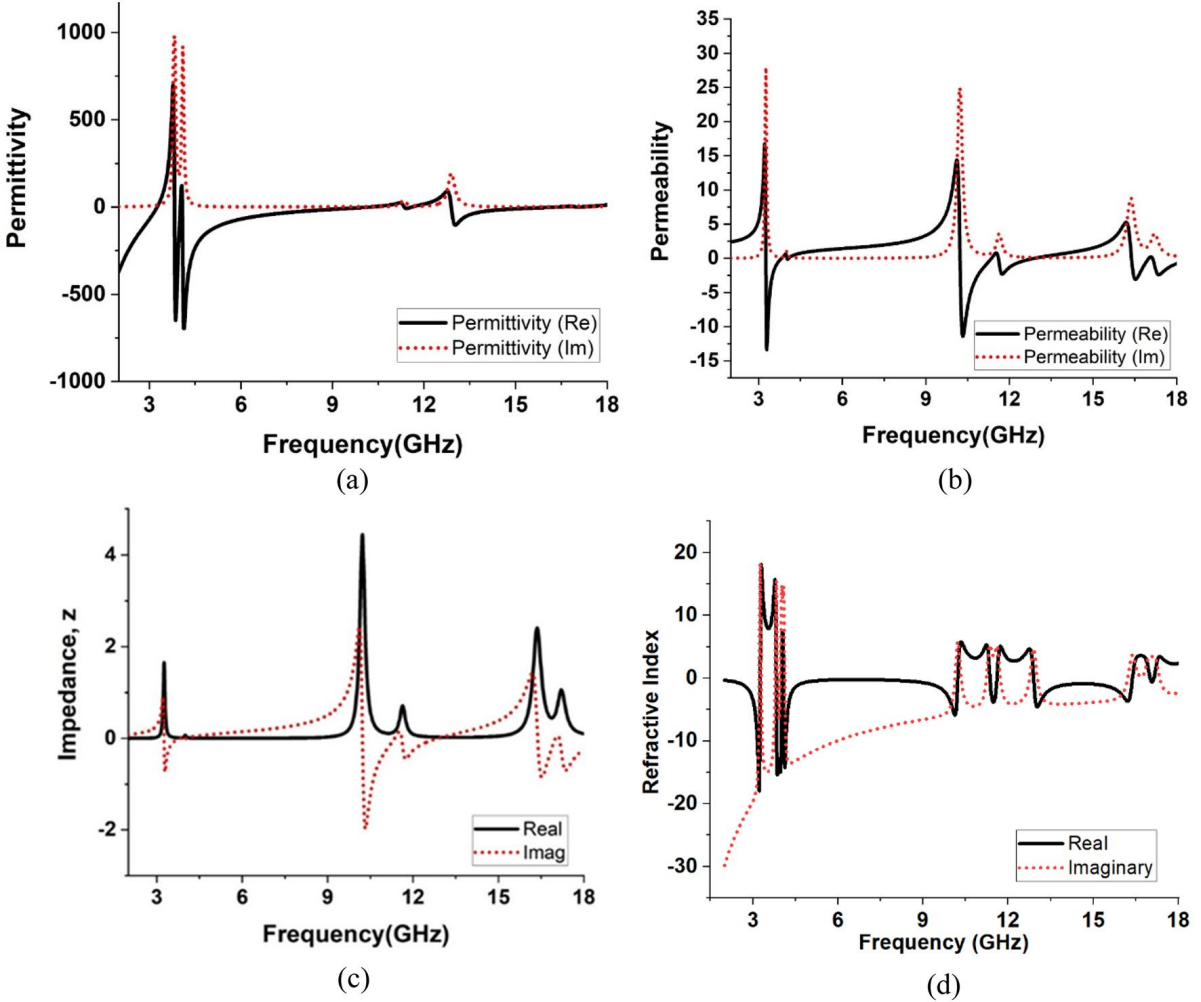
The real and imaginary parts of (**a**) permittivity, (**b**) permeability and (**c**) normalized impedance of the proposed MMA’s unit cell, (**d**) refractive index response of the MMA.

The effective parameters of the proposed MMA, such as permittivity, permeability, and normalized impedance, are presented in Fig. 17 to verify the metamaterial behaviour. The transmission coefficient S_21_ and reflection coefficient S_11_ simulated data are used to compute permittivity and permeability. Equations (7) and (8) are used to calculate the relative permittivity and permeability of the MMA for a surface with a thickness of 1.6 mm. The wave period is considered as *k*_*0*_ = 2*fc*, where *c* stands for light velocity and *f* for microwave signal frequency^46^.

7 $${\text{Permittivity}},\varepsilon_{r} = \frac{2}{{{\text{j}}k_{0} d}} \times \frac{{\left( {1 - S_{11} - S_{21} } \right)}}{{\left( {1{ } + {\text{ S}}_{11} { } + {\text{ S}}_{21} } \right)}}$$

8 $${\text{Permeability}},\mu_{r} = \frac{2}{{{\text{j}}k_{0} d}} \times \frac{{\left( {1 - S_{21} + S_{11} } \right)}}{{\left( {1{ } + {\text{ S}}_{21} { } - {\text{ S}}_{11} } \right)}}$$

The knowledge of relative permittivity and relative permeability is helpful in determining the normalized impedance, *Z* as per Eq. (9).

9 $${\text{Normalized impedance}},{\text{ Z }} = \frac{{{\text{Z}}_{{{\text{eff}}}} { }}}{{{\text{Z}}_{0} }} = \sqrt {{\upmu }_{r} /{\upvarepsilon }_{r} { }}$$

where Z_*eff*_ is the effective impedance that includes combined effects for different portions of the MMA, and Z0 is the free space impedance that is about 377 Ω^24^. The obtained permittivity and permeability are depicted in Fig. 17a, b. From Fig. 17a, it is noticed that relative permittivity becomes negative in the frequency ranges 2–3.24 GHz, 3.8–4.02 GHz, 4.08–10.22 GHz, 11.34–11.6 GHz, 12.88–16.36 GHz, whereas as expressed in Fig. 17b negative permeability is obtained in the frequency ranges 3.24–3.8 GHz, 4.02–4.08 GHz, 10.23–11.34 GHz, 11.6–12.88 GHz, 16.36–18 GHz. This result indicates that the MMA exhibits single negative behaviour in which permittivity and permeability undergo positive to negative transition alternatively. Moreover, absorption peaks are obtained near the frequencies of negative resonance peaks of permeability, indicating that magnetic resonance is a dominating factor for peak absorptions. The normalized input impedance of the MMA cell is shown in Fig. 17c. The extracted data shows that the real parts of the normalized impedance at 3.26 GHz, 11.6 GHz, and 17.13 GHz are 1.56, 0.662, and 0.9, respectively, whereas the imaginary values are − 0.293, − 0.011, and − 0.0072, respectively. An implicit relation between reflection coefficient, MMA impedance, and free space impedance exists and that can be expressed as:$S_{11} = \frac{{Z - Z_{n} }}{{Z + Z_{n} }}$, where normalized impedance of free impedance, *Z*_*n*_ = 1 + *j* 0. The calculated reflection coefficient based on impedance provides absorption around 94%, 95.87%, and 99.7% at 3.26 GHz, 11.6 GHz, and 17.13 GHz, respectively, which are determined using the relation presented in Eq. (1) It is noticed from the extracted data that the mismatch between MMA impedance and free space impedance causes low absorption. As normalized impedance approaches to 1, peak absorption increases towards 100%. Thus, the impedance of the MMA is another important factor to obtain maximum absorption at a particular frequency. For unity absorption, the real part of the normalized impedance will essentially be l, whereas the imaginary part will be zero. Figure 17d exhibits the refractive response of the proposed model. From Fig. 17d, it is identified that the response is negative in the peak resonance frequency. The refractive index is related to the SNG or DNG property of the unit cell. For DNG property, both permittivity and permeability should be negative, resulting in a positive refractive index. It is noticed that the refractive index becomes negative in the frequency ranges 2–3.29 GHz, 3.77–3.82 GHz, 4.12–4.48 GHz, 8.03–10.12 GHz, 11.36–11.6 GHz, 12.91–16.35 GHz and 17.02–17.12 GHz. The proposed MMA exhibits an SNG behaviour because of the permittivity and permeability from positive to negative with a negative refractive index.

### Polarization insensitive behaviour of MMA

**Figure 18:**
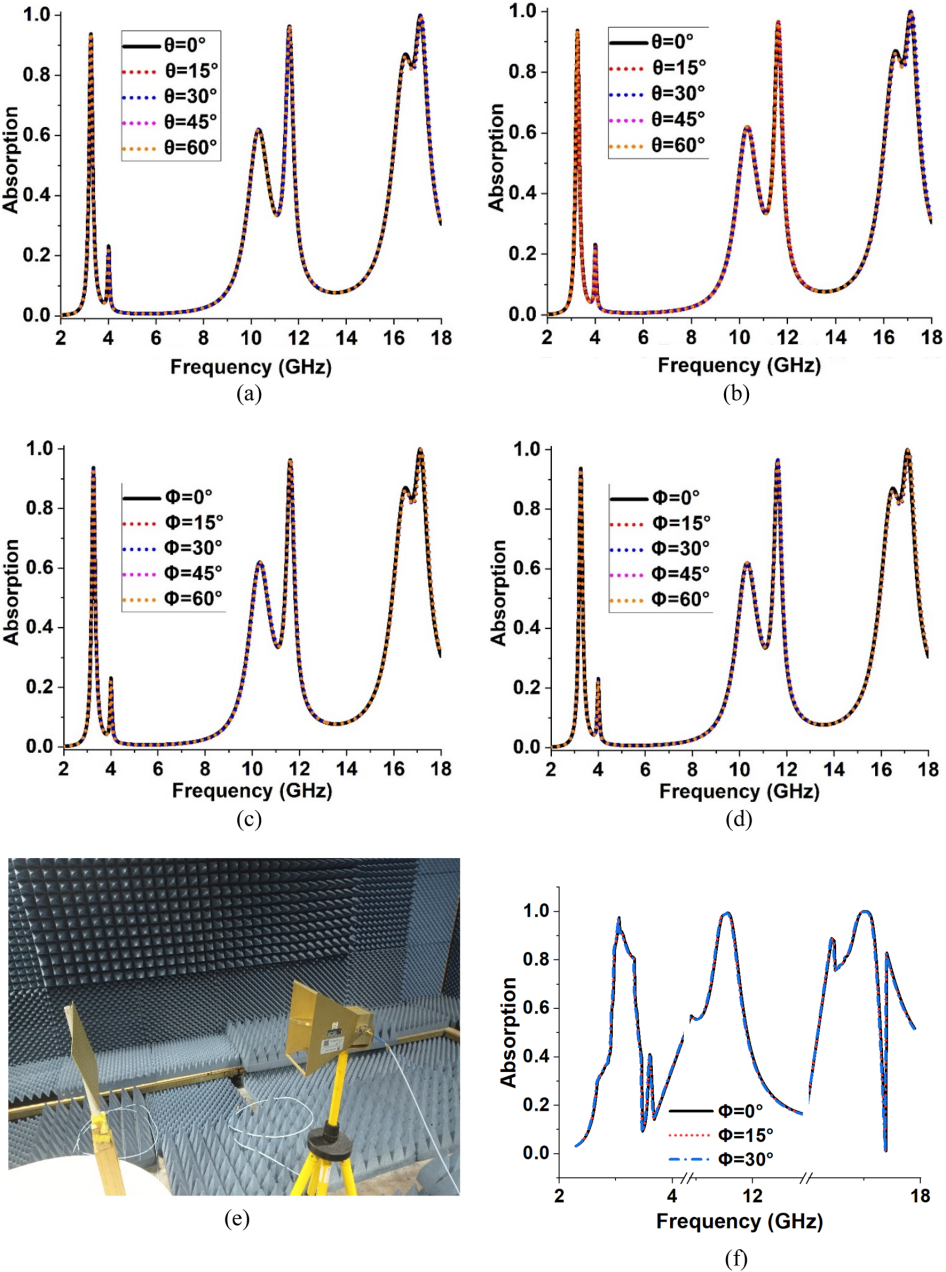
Apsorption for : (**a**) incident angle (θ) change at TE mode, (**b**) incident angle (θ) change at TM mode, (**c**) polarization angle (Φ) change at TE mode, (**d**) polarization angle (Φ) change at TM mode, (**e**) measurement set-up for polarization anlge, and (**f**) Measurement results of polarization angle (Φ).

The polarization-insensitive performance of the absorber for various incident and polarization angles of the incoming electromagnetic waves identifies the perfect absorption of the metamaterial absorber. The polarization-insensitive characteristic of the absorber depends on the structure of the resonator and the thickness of the substrate material. The response to TE and TM waves monitors the effect of the angle variation. For the simulation of MMA, Floquet ports are utilized. The boundaries of the unit cell are selected in the X and Y-axis for electric and magnetic fields, respectively, whereas the polarized signal is moved in the Z direction. The incident angle (θ) and polarization angle (Φ) variation of 15°, 30°, 45°and 60° are used for the polarized singles of TE and TM modes. The absorption spectrums for incident angle variations are plotted in Fig. 18a and b for TM and TE modes, respectively, whereas the absorption spectrums for polarization angle variations are shown in Fig. 18c and d. It is observed that for both TE and TM modes, the peak absorptions and the bandwidths for five different incident and polarization angles are not hampered, which can be indicated by Fig. 18a–d, respectively. Figure 18e described the measurement set-up for the polarization angle in the anechoic chamber from the angle 0^ο^–30^ο^. The experiment results also exhibit similar results at the variation of different angles as shown in Fig. 18f. So, the proposed MMA has a polarization-insensitive characteristic. The symmetric structure of the MMA is the main reason behind this polarization-insensitive behaviour as also indicated in^47^.

### Testing of proposed MMA design

**Figure 19:**
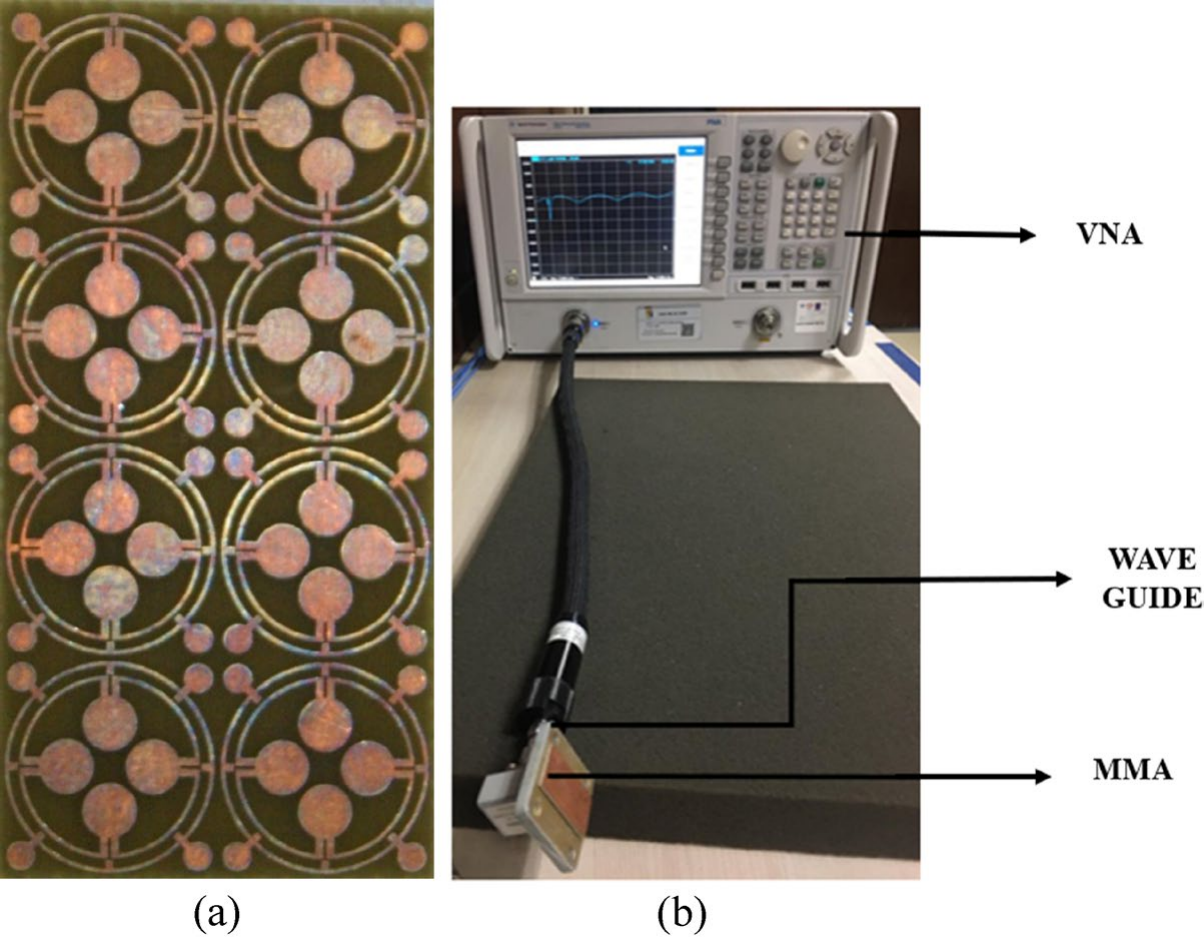
(**a**) prototype 4 × 2 array of the MMA unit cell, (**b**) measurement set-up.

**Figure 20:**
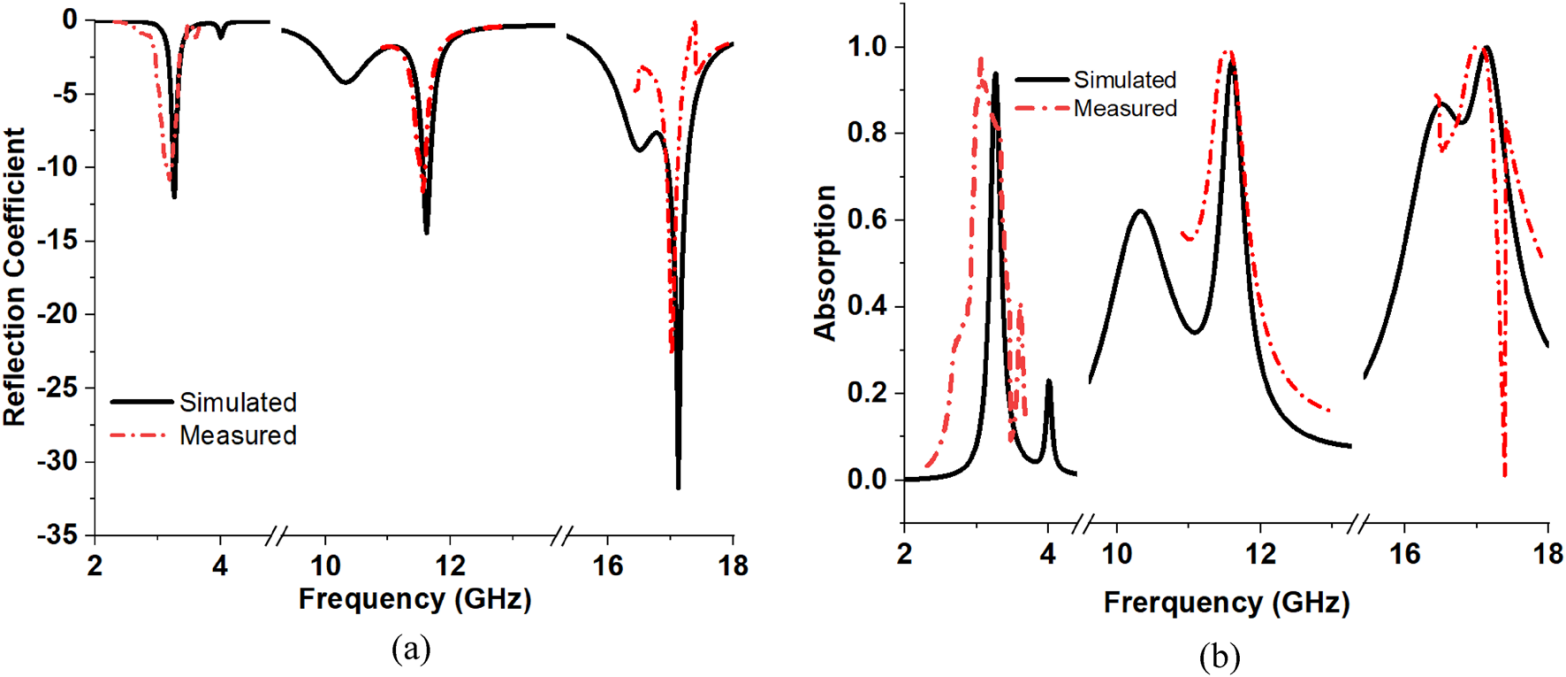
Simulation and measurement results of the MMA (**a**) reflection coefficient response, (**b**) absorption response.

The prototype of the array of the proposed MMA unit cell is developed, and measurement data is taken for S_11_ and S_21_ using a vector network analyser (VNA). The fabricated prototype of the 4 × 2 array is presented in Fig. 19a, and the measurement set-up is depicted in Fig. 19b. Before fabrication, the array performance is checked in simulation, and it is found that the array of the unit cells provides a result that is similar to that of the unit cell. For brevity, the simulated result is not presented here. A similar result is obtained due to the symmetrical structure of the proposed MMA. Since array and unit cell performance are the same, the experiment is performed using the array. In the measurement set up as shown in Fig. 19b, a waveguide adapter is connected to the VNA port using a coaxial cable, and the prototype is placed on the waveguide adapter. Two types of waveguide ports are used for the measurements of the MMA. For the S-band measurement, we used an 85 mm × 45 mm dimension waveguide, and for the X and Ku band, we used a 20 mm × 10 mm dimension waveguide. Since MMA uses full copper at the back side, the transmission is reduced to zero, and using VNA, the reflection coefficient is measured. Figure 20a presents a comparison value of S_11_ in terms of simulation and measurement. The measured S_11_ is used to calculate absorption using the formula of Eq. (1). Thus, the measured absorption is obtained, and it is compared with the simulation result using the plots presented in Fig. 20b. From the simulation, three-peak absorption of 93.8%, 96.74%, and 99.95% are attained at 3.26 GHz, 11.6 GHz, and 17.13 GHz, respectively, as shown in Fig. 20. Whereas, the peak absorptions of 97.85%, 99.21% and 99.87% are obtained at 3.05 GHz, 11.504 GHz, and 16.988 GHz, respectively for the measurement set-up as shown in Fig. 20. There is a small deviation between simulation and measurement results. The first resonance frequency of the measurement was reduced by 6.44% from the simulation but had an increased absorption of 4.31%. The second resonance frequency of the measurement is also reduced by 0.82%, with a peak absorption improvement of 2.55% compared with the simulation. Similarly, the third resonance frequency was reduced by 0.85% to provide a decreased peak absorption of 0.08% from the simulation. Ignoring these small deviations between the simulation and measurement results of the absorber, it can be concluded that there is a good agreement between simulation and measurement results. The first resonance peak from measurements is at 3.05, which is close to the simulation one because of the FR-4 substrate, which performs well within the 10 GHz frequency range. For this reason, there is a close proximity between simulation results and measured results. As the frequency increased from 10 GHz the dielectric loss of the FR-4 started to increase, and the permittivity started to decrease with the frequency. For this reason, there is a small deviation between simulation and measurement results. However, as the resonance occurs at 16.988 GHz, there is a significant amount of dissimilarity between simulation and measurement results due to high dielectric loss.

The mismatch of the measured result may be incurred due to fabrication errors, loss in the coaxial cables, and coupling effect between the waveguide adapter and coaxial cable as well as waveguide adapter and MMA array, unnecessary radioactivity from the environment, faults in VNA measurements, crystal deficiencies, the impact of fringing, signal leakage. The air gap between the waveguide due to fitness mismatch may affect the performance of a metamaterial absorber by introducing undesired reflections into the measuring environment. It is important to make sure that the waveguide used for testing has the same size and shape as the sample. Also, calibration can improve the results if there are any undesired errors. Two common ways to calibrate are through short-open-load-through (SOLT) calibration or using calibration kits. In addition, from Fig. 20, it is also observed that with the increase in frequency to the higher order for both simulation and measurement, the bandwidths of the resonance frequencies increase significantly. The reason behind this is the use of FR4 material as the substrate of the absorber, which has high absorption loss at higher frequencies.

### Liquid sensing procedure using the proposed MMA

**Figure 21:**
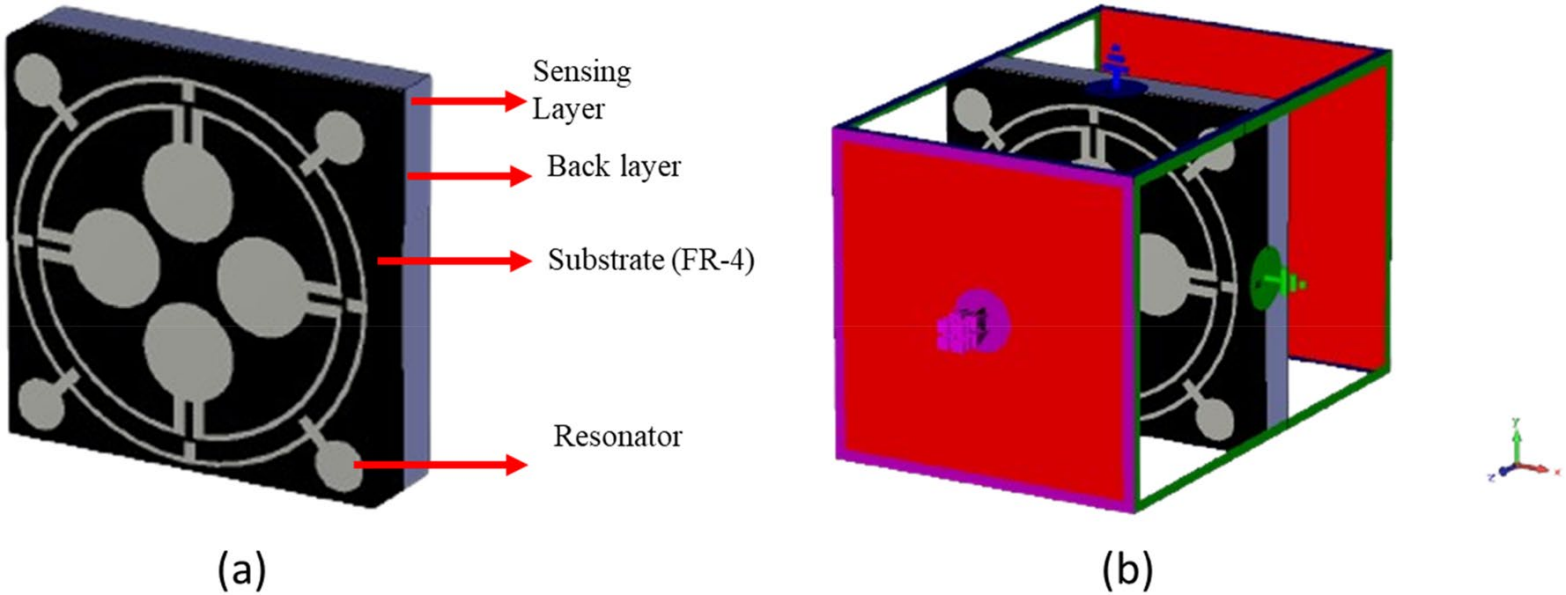
(**a**) Construction of the proposed unit cell’s sensing arrangement. (**b**) Simulation set-up for the sensing application.

**Figure 22:**
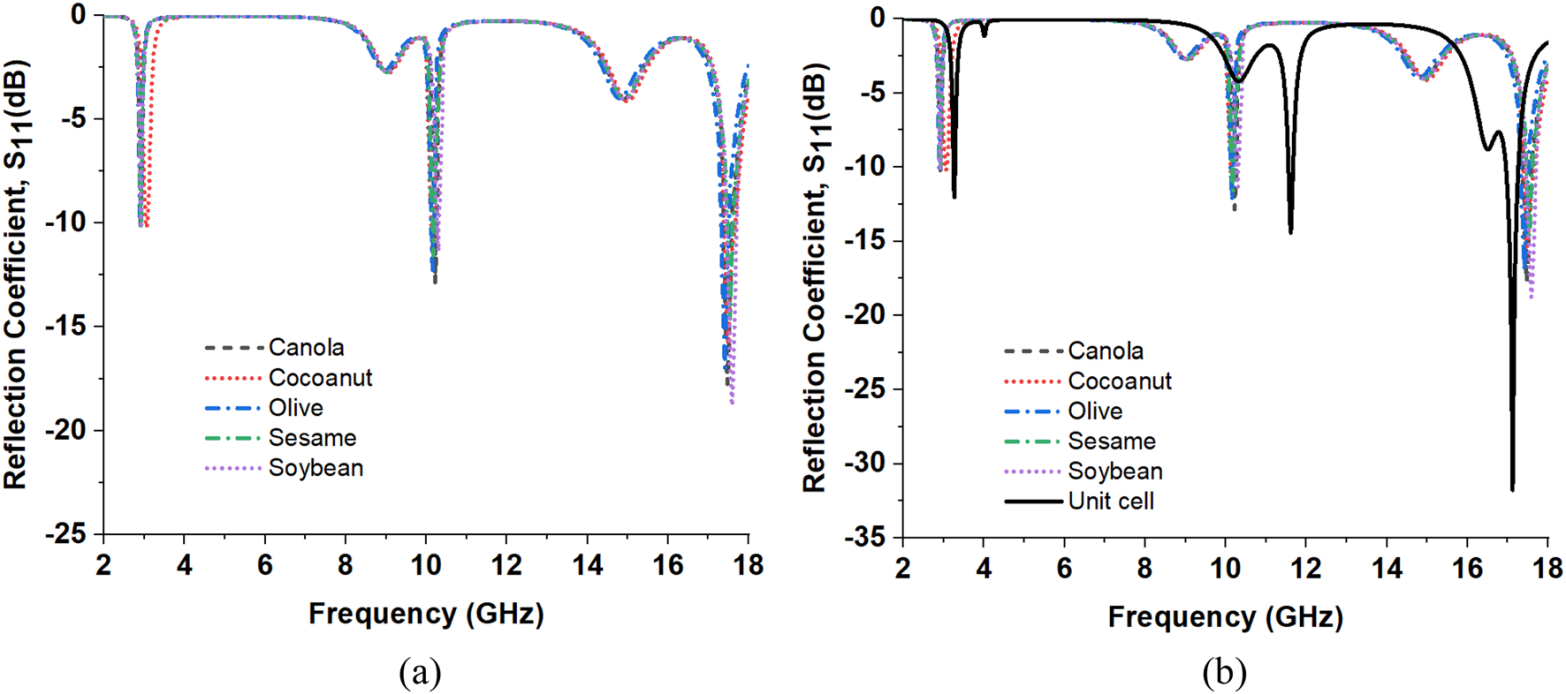
Observation of the Sensor’s reflection coefficient response. (**a**) The reflection coefficient response for various oil sample types. (**b**) Comparison of the Sensor’s S11 response with the S11 response of the normal absorber.

Substrate thickness and material dielectric property are variables that can be adjusted to modify the MMA property. The sensing performance of MMA is consequently determined by the variation of the dielectric constant. There are two ways that MMAs can be used for sensing: either insert the Sensor between the patch substrate and the bottom of the sensor substrate or add a sensor layer to the MMA patch^48,49^. The sensing capability we have presented can function as a liquid sensor. The proposed MMA sensor can be purposefully constructed in such a way as to interact productively with specific frequencies of electromagnetic radiation. The reverse side of the copper layer is coated with a 1 mm sensing layer, as illustrated in Fig. 21a of the sensing application design. Figure 21b describes the overall simulation set-up of the Sensor. A comparative analysis is performed to assess the sensing capabilities of five different oils: (1) Olive oil (dielectric constant 3.03), (2) Sesame oil (dielectric constant 2.91), (3) Coconut oil (dielectric constant 2.82), (4) Soybean oil (dielectric constant 2.9), and (5) Canola oil (dielectric constant 2.93). Figure 22a presents a comparison between the MMA’s S_11_ value and the Sensor’s S_11_ value. Meanwhile, Fig. 22b illustrates the reaction of the Sensor’s reflection coefficient. From Eq. (4), it can be concluded that the Dielectric constant has a direct relation with the reflection coefficient. When the dielectric constant value changes, it has a direct effect on the S_11_ response and also changes the absorption response of the MMA according to the S_11_ response, as absorption is dependent on the reflection coefficient.

Operating frequencies for the MMA absorber are 3.26 GHz, 11.6%, and 17.13 GHz. The frequencies mentioned indicate the maximum reflection coefficient and maximal absorption, respectively.

**Figure 23:**
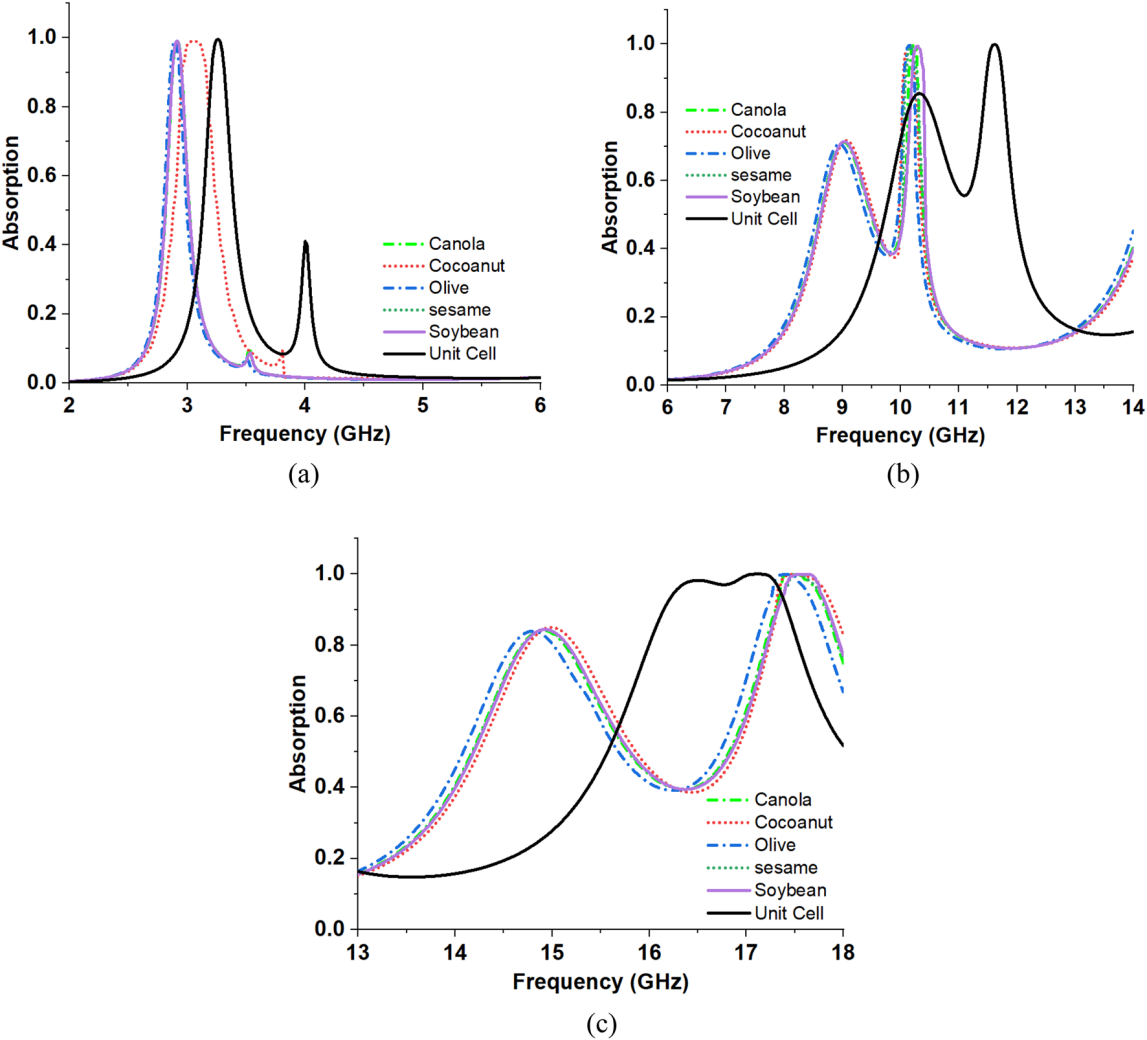
Absorption response of the Sensor at different oil samples. (**a**) S band response. (**b**) X band response. (**c**) Ku band response.

Various oils with distinct dielectric constants were introduced into the air gap, ranging from 2.8 to 3.1. The absorption curve of the MMA is altered by the varying dielectric constants of the oil samples employed in the sensing layer. Consequently, the absorption of the two lower bands and one higher band in the quad-band decreases. Figure 23 displays the absorption graphs for several oil compounds. Figure 23 depicts an enlarged view of the absorption peaks, aiming to enhance comprehension of the resonant frequency shift in relation to the dielectric constant.

Figure 23 describes the Sensor’s absorption response according to the different samples of the oils. From Fig. 23, frequency shifting scenarios are extracted. For different dielectric constants, the resonance peaks for the S-band (2–4 GHz) frequency range are at 2.91 GHz, 3.04 GHz, 2.89 GHz, 2.90 GHz, 2.41 GHz and 2.91 GHz for canola oil, coconut oil, olive oil, sesame oil, and soybean oil respectively. In these cases, when the dielectric constant value increased, the resonance came closer to the lower frequency. The frequency shift in the X band (8–12 GHz) region with the resonance peaks of 10.24 GHz, 10.14 GHz, 10.16 GHz, 10.16 GHz, and 10.27 GHz. Other frequency-shifting resonance peaks for the Ku band (12–18 GHz) region are at 17.50 GHz, 17.48 GHz, 17.42 GHz, 17.58 GHz, and 17.58 GHz for canola oil, coconut oil, olive oil, sesame oil, and soybean oil respectively. For a higher dielectric constant value, the resonance peaks go right to the graph, which means they have a higher frequency; for a lower dielectric constant value, the resonance peaks go left to the graph, representing a lower frequency. Figure 23a, and b describe that for the frequency range of S and X bands, the frequency shift is on the lower frequency side, which means the shift is decreased, but on the other hand, at the Ku band frequency range, the shift is increased at the higher frequency side. From the frequency range in the S, X, and Ku bands, the magnitude change is also monitored from the diagram. For the different dielectric constant-based oil samples have a significant influence on the magnitude level. So, this Sensor can work by changing frequency.

### Comparison

The proposed MMA is compared with some recent relevant research works, as presented in Table 7. Table 7 compares the size, resonance frequencies, bandwidth, Polarization Insensitivity, peak absorption, Effective medium ratio (EMR), and coverage bands of the introduced MMA structure compared to several current publications regarding the MM structure. The compactness of the MM can also be measured by comparing the structure's dimensions to the wavelength using the relation EMR = λ/L, where λ is the wavelength determined at the smallest frequency of resonance and L is the largest dimension. High EMR enables precise field rationing and mirror symmetry during design. A unit cell with a high EMR value increases homogeneity and electrical capacity without limitations on fabrication. The dimension of the unit cells of^50–52^ is smaller compared to our proposed model, whereas^38,53^ used larger dimensions for their designed absorbers. The low-cost FR4 material is used as the substrate in all the models presented in the references of^30–34^, including our proposed model. Due to the asymmetrical structure, the model of reference^30^ is sensitive to both zenith and azimuth angles. The absorber models of^32,34^ claimed the polarization insensitive behaviour, but after careful observation, it was found that their models have variation in absorption for zenith and azimuth angles. The absorber of^31^ showed polarization-insensitive behaviour. Our proposed model can also display polarization-insensitive behaviour up to 60° because of its symmetrical structure. The absorber models^30,32,33^ presented in the table are designed for two frequency bands, whereas^31,34^ can cover a single frequency band, but all the designs have small EMR values. The models of references^39,40^ offer sensing operation in single-band sensing. These designs lack sensing flexibility to operate in multiband and also have lower EMR values with high dimensions. On the other hand, our proposed model can provide peak absorptions for three different frequency bands with a higher EMR value.

**Table 7:** Comparing the suggested MMA cell’s performance with that of existing state-of-the-art MMAs.

References	Substrate	Physical dimension (mm)	Resonances frequency (GHz)	Polarization insensitive	Peak absorption (%)	Frequency band	EMR	Remarks
^30^	FR4	8 × 8	15.52, 27.24	No	98.38%, 80.07%	Ku, Ka band	2.41	Energy harvesting
^31^	FR4	10 × 10	12 to 18	Yes	above 88%	Ku band	2.5	broadband absorber
^32^	FR4	10 × 10	11.4, 17.1, 19.2	Yes	above 90%	X, Ku, K band	2.63	Flexible Absorber
^33^	FR4	20 × 20	13.78, 15.3	Not specified	99.6% and 99.14%	Ku band	1.08	Energy harvesting
^34^	FR4	15.6 × 15.6	4.0 to 8.0	Yes	above 90%	C band	4.80	C and X band application
^39^	Rogerscorp	35 × 35	6.46, 7.68	Not specified	Above 90%	C band	1.32	pressure, temperature, density, and humidity sensing
^40^	FR4	20 × 20	8 to 13	Yes	77.1–99.9%	X band	1.76	metamaterial-based Sensor
Proposed	FR4	12 × 12	3.26, 11.6, 17.13	Yes	93.8, 96.74, 99.95	S, X, Ku band	7.66	Absorber with liquid sensing

## Conclusion

This article presents a new metamaterial (MMA) with a circular ring-based resonator and dumbbell-shaped structure attachment. The MMA is designed over an FR-4 substrate and provides 93.8%, 96.74%, and 99.95% absorption at 3.26 GHz, 11.6 GHz, and 17.13 GHz for S, X and Ku band sensing applications. The prototype demonstrates similar results to the simulated results, showing high Q-factors at targeted frequencies. The study analyzes the performance of MMA for transverse electric and transverse magnetic modes of electromagnetic waves. It shows similar responses for different incident angles and polarization angles. The resonance phenomena are also examined through equivalent circuit modelling in ADS software, which provides insight into the electrical behaviour of the MMA. Moreover, absorption phenomena are scrutinized through circuit current analysis. The metamaterial’s compact dimension, high Q-factor, and good absorption make it a potential candidate for miniaturized microwave devices, particularly for sensing and detecting purposes. By utilising the absorption response of the model, this article describes a high-sensitivity sensing application that can differentiate different kinds of oil.

## Data Availability

The datasets deployed and analysed in the course of this research are not accessible to the general public as per the regulations of Universiti Kebangsaan Malaysia (affiliated institution). However, they are accessible upon adequate request from the corresponding author in order to facilitate further research.

## References

[1] Engheta N, Ziolkowski RW (2006). Metamaterials: Physics and Engineering Explorations.

[2] Mehrabi S, Bilal RMH, Naveed MA, Ali MM (2022). Ultra-broadband nanostructured metamaterial absorber based on stacked square-layers of TiN/TiO 2. Opt. Mater. Express.

[3] Naveed MA, Bilal RMH, Baqir MA, Bashir MM, Ali MM, Rahim AA (2021). Ultrawideband fractal metamaterial absorber made of nickel operating in the UV to IR spectrum. Opt. Express.

[4] Khalil MA (2023). Liquid chemical adulteration detection enhancement using a square enclosed Tri-Circle negative index metamaterial sensor. Eng. Sci. Technol. Int. J..

[5] Montoya JA, Tian Z-B, Krishna S, Padilla WJJOE (2017). Ultra-thin infrared metamaterial detector for multicolor imaging applications. Opt. Express.

[6] Hossain MB, Faruque MRI, Islam MT, Singh M, Jusoh MJJOMR (2022). Triple band microwave metamaterial absorber based on double E-shaped symmetric split ring resonators for EMI shielding and stealth applications. J. Mater. Res. Technol..

[7] Hasan MS (2023). A symmetric plus-shape resonator based dual band perfect metamaterial absorber for Ku band Wireless Applications. Opt. Mater..

[8] Rabbani MG (2024). Orthogonal centre ring field optimization triple-band metamaterial absorber with sensing application. Eng. Sci. Technol. Int. J..

[9] Tran HN, Nguyen VH, Nguyen BH, Vu DL (2016). Light trapping and plasmonic enhancement in silicon, dye-sensitized and titania solar cells. Adv. Nat. Sci.: Nanosci. Nanotechnol..

[10] Mulla B, Sabah C (2016). Multiband metamaterial absorber design based on plasmonic resonances for solar energy harvesting. Plasmonics.

[11] Hasan MS (2024). Double elliptical resonator based quad-band incident angle and polarization angle insensitive metamaterial absorber for wireless applications. Opt. Laser Technol..

[12] Landy NI, Sajuyigbe S, Mock JJ, Smith DR, Padilla WJ (2008). Perfect metamaterial absorber. Phys. Rev. Lett..

[13] Lian Y (2016). Dual-band near-infrared plasmonic perfect absorber assisted by strong coupling between bright-dark nanoresonators. Opt. Commun..

[14] Wang B-X (2016). Quad-band terahertz metamaterial absorber based on the combining of the dipole and quadrupole resonances of two SRRs. IEEE J. Sel. Top. Quant. Electron..

[15] Hu D, Wang H, Tang Z, Zhang X, Zhu Q (2016). Design of four-band terahertz perfect absorber based on a simple#-shaped metamaterial resonator. Appl. Phys. A.

[16] Misran N, Yusop SH, Islam MT, Ismail MY (2012). Analysis of parameterization substrate thickness and permittivity for concentric split ring square reflectarray element. J. Eng..

[17] Alam A, Islam SS, Islam MH, Almutairi AF, Islam MT (2020). Polarization-independent ultra-wideband metamaterial absorber for solar harvesting at infrared regime. Materials.

[18] Lou P, Wang B-X, He Y, Tang C, Niu Q, Pi F (2020). Simplified design of quad-band terahertz absorber based on periodic closed-ring resonator. Plasmonics.

[19] Islam M, Islam MT, Moniruzzaman M, Samsuzzaman M, Arshad H (2021). Penta band single negative meta-atom absorber designed on square enclosed star-shaped modified split ring resonator for S-, C-, X-and Ku-bands microwave applications. Sci. Rep..

[20] Islam MS, Samsuzzaman M, Beng GK, Misran N, Amin N, Islam MT (2020). A gap coupled hexagonal split ring resonator based metamaterial for S-band and X-band microwave applications. IEEE Access.

[21] Alkurt FO (2018). Octagonal shaped metamaterial absorber based energy harvester. Mater. Sci..

[22] Ünal E, Bağmancı M, Karaaslan M, Akgol O, Arat HT, Sabah C (2017). Zinc oxide–tungsten-based pyramids in construction of ultra-broadband metamaterial absorber for solar energy harvesting. IET Optoelectron..

[23] Elsharabasy A, Bakr M, Deen MJ (2020). Wide-angle, wide-band, polarization-insensitive metamaterial absorber for thermal energy harvesting. Sci. Rep..

[24] Moniruzzaman M, Islam MT, Muhammad G, Singh MSJ, Samsuzzaman M (2020). Quad band metamaterial absorber based on asymmetric circular split ring resonator for multiband microwave applications. Results Phys..

[25] Moniruzzaman M, Islam MT, Mansor MF, Soliman MS, Misran N, Samsuzzaman M (2023). Tuning metallic stub loaded symmetrical resonator based dual band metamaterial absorber for wave shielding from Wi-Fi frequencies. Alexandr. Eng. J..

[26] Nguyen TKT (2021). Simple design of a wideband and wide-angle insensitive metamaterial absorber using lumped resistors for X-and Ku-bands. IEEE Photon. J..

[27] Singh H, Sharma A, Gupta A, Singhal AJM, Letters OT (2024). A polarization-insensitive metamaterial absorber for moisture-sensing applications of agriculture products. Microwave Opt. Technol. Lett..

[28] Roy K, Sinha R, Barde CJF (2022). Linear-to-linear polarization conversion using metasurface for X, Ku and K band applications. Frequenz.

[29] Ranjan P, Barde C, Choubey A, Sinha R, Mahto SKJSAS (2020). Wide band polarization insensitive metamaterial absorber using lumped resistors. SN Appl. Sci..

[30] Wei Y (2022). A multiband, polarization-controlled metasurface absorber for electromagnetic energy harvesting and wireless power transfer. IEEE Trans. Microwave Theory Tech..

[31] Wu Y, Wang J, Lai S, Zhu X, Gu W (2018). Transparent and flexible broadband absorber for the sub-6G band of 5G mobile communication. Opt. Mater. Express.

[32] Wu Y, Wang J, Lai S, Zhu X, Gu W (2019). A transparent and flexible microwave absorber covering the whole WiFi waveband. AIP Adv..

[33] Cheng Y, Luo H, Chen F (2020). Broadband metamaterial microwave absorber based on asymmetric sectional resonator structures. J. Appl. Phys..

[34] Ranjan P, Barde C, Choubey A, Sinha R, Jain A, Roy KJF (2022). A wideband metamaterial cross polarizer conversion for C and X band applications. Frequenz.

[35] Cao H, Shan M, Chen T, Lei J, Yang L, Tan X (2019). Triple-band polarization-independent ultrathin metamaterial absorber. Progress Electromagn. Res. M.

[36] Zhou Q (2019). Optically transparent and flexible broadband microwave metamaterial absorber with sandwich structure. Appl. Phys. A.

[37] Ranjan P (2022). The synthesis of a pixelated metamaterial cross-polarizer using the binary wind-driven optimization algorithm. J. Comput. Electron..

[38] Hoque A, Tariqul-Islam M, Almutairi AF, Alam T, Jit-Singh M, Amin NJS (2018). A polarization independent quasi-TEM metamaterial absorber for X and Ku band sensing applications. Sensors.

[39] Bakır M, Karaaslan M, Unal E, Akgol O, Sabah CJO-ER (2017). Microwave metamaterial absorber for sensing applications. Opto-Electron. Rev..

[40] Lateef OS, Al-Badri M, Al-Badri KSL, Mohammed SAJSR (2023). Polarization-insensitive Archimedes’-spiral-shaped ultrathin metamaterial absorbers for microwave sensing application. Sci. Rep..

[41] Bakır M, Karaaslan M, Dinçer F, Delihacioglu K, Sabah CJJ (2016). Tunable perfect metamaterial absorber and sensor applications. J. Mater. Sci. Mater. Electron..

[42] Pozar DM (2011). Microwave Engineering.

[43] Liu N, Giessen H (2010). Coupling effects in optical metamaterials. Angew. Chem. Int. Ed..

[44] Gay-Balmaz MO (2002). Electromagnetic resonances in individual and coupled split-ring resonators. J. Appl. Phys..

[45] Raab B (2003). "Lumped elements for RF and microwave circuits. Microwave J..

[46] Ziolkowski RW (2003). Design, fabrication, and testing of double negative metamaterials. IEEE Trans. Antennas Propag..

[47] Nguyen TT, Lim S (2018). Design of metamaterial absorber using eight-resistive-arm cell for simultaneous broadband and wide-incidence-angle absorption. Sci. Rep..

[48] Islam MR (2021). Tri circle split ring resonator shaped metamaterial with mathematical modeling for oil concentration sensing. IEEE Access.

[49] Zhang Y, Zhao J, Cao J, Mao BJS (2018). Microwave metamaterial absorber for non-destructive sensing applications of grain. Sensors.

[50] Zhao G, Bi S, Cui Y (2019). Study on the characteristics of a V-shaped metamaterial absorber and its application. AIP Adv..

[51] Barde C, Choubey A, Sinha R, Mahto SK, Ranjan P (2020). A compact wideband metamaterial absorber for Ku band applications. J. Mater. Sci. Mater. Electron..

[52] Lakshmi M, Prasad-Jones-Christydass S, Kannadhasan S, Anguraj K, Chatterjee JM (2023). Polarization stable triband thin square-shaped metamaterial absorber. Int. J. Antennas Propag..

[53] Hoa NTQ, Tuan TS, Hieu LT, Giang BL (2019). RETRACTED ARTICLE: Facile design of an ultra-thin broadband metamaterial absorber for C-band applications. Sci. Rep..

